# Next-Generation Sequencing for Infectious Disease Diagnostics in Pediatric Patients with Malignancies or After Hematopoietic Cell Transplantation: A Systematic Review

**DOI:** 10.3390/jcm14186444

**Published:** 2025-09-12

**Authors:** Anna Jabłońska, Aleksander Sadkowski, Monika Richert-Przygońska, Jan Styczyński

**Affiliations:** 1 Doctoral School of Medical and Health Sciences, Ludwik Rydygier Collegium Medicum, Nicolaus Copernicus University, 85-067 Bydgoszcz, Poland; 2 Department of Pediatric Hematology and Oncology, Ludwik Rydygier Collegium Medicum, Nicolaus Copernicus University, 85-094 Bydgoszcz, Poland; monika_richert@yahoo.com (M.R.-P.); jstyczynski@cm.umk.pl (J.S.); 3 Student Scientific Society, Ludwik Rydygier Collegium Medicum, Nicolaus Copernicus University, 85-067 Bydgoszcz, Poland; sadkowskialeksander80@gmail.com

**Keywords:** next-generation sequencing, metagenomic sequencing, pediatric oncology, hematopoietic cell transplantation, infectious disease, immunocompromised children

## Abstract

**Background**: Immunocompromised children with malignancies or after hematopoietic cell transplantation (HCT) often deteriorate before conventional cultures identify a pathogen. Next-generation sequencing (NGS) promises faster, broader detection, yet its clinical impact in pediatric oncology remains unclear. This review aimed to assess the diagnostic performance and clinical utility of NGS in this population. **Methods**: We searched PubMed, Embase, and Scopus from January 2010 to April 2025 for studies evaluating NGS (metagenomic, targeted, or whole-genome sequencing) in pediatric oncology or HCT patients meeting predefined eligibility criteria. Duplicate screening, data extraction, and Joanna Briggs Institute risk-of-bias appraisal were performed. Heterogeneity precluded formal meta-analysis; findings were synthesized using narrative synthesis complemented by limited quantitative analyses. The protocol was not registered. **Results**: Twenty-four studies (≥2700 children; 2019–2025) met inclusion criteria. Metagenomic NGS (mNGS) was the most common approach, applied to blood/plasma (46%), bronchoalveolar fluid (BALF) (21%), and other fluids. In culture-negative sepsis or persistent febrile neutropenia, mNGS detected pathogens in 69–86% of episodes versus 18–56% for culture/polymerase chain reaction (PCR). Described in limited studies, early (<48 h) testing shortened fever by ~1.5 days and cut antimicrobial costs by 25–30%. Across studies, treatment was escalated, de-escalated, or discontinued in a median of 63% of mNGS-positive cases. Whole-genome sequencing (WGS) identified 18 silent transmission clusters and resolved a multidrug-resistant *Acinetobacter baumannii* outbreak within hours. **Conclusions**: NGS benefits pediatric hemato-oncology by accelerating pathogen-directed therapy, supporting antimicrobial stewardship, and enhancing outbreak surveillance. Despite cost and standardization barriers, evidence supports its use in selected high-risk patients.

## 1. Introduction

Infections are a common cause of morbidity and mortality among pediatric patients with malignancies. Immunocompromised individuals are at a heightened risk of contracting severe viral, bacterial, and fungal infections due to impaired innate and adaptive immune responses, neutropenia, compromised mucosal barriers, the presence of indwelling central venous catheters (CVCs), and exposure to the healthcare environment [1,2]. Prompt identification of the causative agent and early access to treatment are essential for the timely initiation of targeted therapy [3]. This targeted approach not only enhances patient outcomes but also minimizes the reliance on broad-spectrum treatments [3,4].

Traditional diagnostic techniques, including culture, nucleic acid amplification tests, cell culture-based assays, and innovative technologies like syndromic multiplex polymerase chain reaction (PCR), have several limitations, including limited sensitivity, specificity, and efficiency, and often require at least 48 h to detect commonly encountered pathogens [3,5]. Moreover, these methods rely on a clinician’s initial suspicion of the specific pathogen involved; their effectiveness is compromised in cases of atypical presentations or unclear causes, as these infections can be caused by a wide range of pathogens, necessitating testing with multiple diagnostic panels. Notwithstanding these advancements, the underlying causes of many infectious diseases remain unclear [5,6].

Massively parallel or high-throughput sequencing, also known as next-generation sequencing (NGS), has become a highly effective tool for comprehensive microbial identification. NGS encompasses various technologies capable of sequencing thousands to billions of DNA and RNA fragments simultaneously and independently. The applications of NGS in clinical pathogen detection are diverse and include metagenomic NGS (mNGS), which enables the direct identification of pathogens, regardless of their type (viruses, bacteria, fungi, and parasites), from clinical samples without the need for prior microbial testing or culturing [7]. This approach makes it possible to replace multiple traditional diagnostic tests with a single mNGS test [5,8].

NGS also enables the identification of antimicrobial resistance genes and mutations, thereby facilitating optimal therapy selection. Research has shown that NGS surpasses traditional diagnostic methods in sensitivity and range of pathogen detection, particularly when standard tests fail to identify rare, novel, or difficult-to-detect pathogens [8,9,10]. NGS shows substantial potential to enhance diagnostic precision and inform more effective treatment strategies, ultimately improving outcomes in pediatric oncology [4]. This systematic review provides a comprehensive evaluation of the application, diagnostic performance, and clinical impact of next-generation sequencing technologies in detecting infectious diseases in pediatric patients with malignancies or after hematopoietic cell transplantation. It addresses the clinical question of how, in this population (P), NGS-based approaches (I) compare with conventional microbiological methods (C) in identifying infectious agents, improving diagnostic yield, and influencing clinical outcomes (O).

## 2. Overview of Next-Generation Sequencing Approaches in Infectious Disease Diagnostics

Next-generation sequencing encompasses a range of techniques that enable rapid sequencing of nucleic acids to identify infectious pathogens. Multiple NGS-based methods have been developed and adapted for clinical diagnostics, each offering distinct applications and advantages.

NGS is a powerful tool for detecting genetic variants and mutations in DNA and RNA. This technology integrates different sequencing chemistries, platforms, and bioinformatics tools. In a comparatively short amount of time, this combination enables enormous parallel sequencing of different lengths of DNA or RNA sequences, or even an entire genome [11]. In microbiology, NGS is primarily useful for replacing traditional pathogen characterization based on morphology, staining characteristics, and metabolic criteria with a genomic description of pathogens [12]. These NGS techniques provide culture-independent detection of microorganisms that are difficult to cultivate [4]. Numerous other fields of infectious disease research are also utilizing NGS technology, including tracking the origins of pathogens, assessing the microbial makeup of host habitats, and testing viruses or cultured isolates for medication resistance [7].

### 2.1. Metagenomic Next-Generation Sequencing

mNGS is a technique that enables parallel sequencing of all nucleic acids (DNA and/or RNA) present in a clinical specimen. This process involves separating and amplifying both host and pathogen nucleic acids from the sample [5]. Applications in infectious disease diagnostics include direct identification of microorganisms from primary clinical samples, prediction of antimicrobial and antiviral resistance via resistance gene characterization, and identification of virulence determinants (e.g., endotoxin or exotoxin secretion) at the species or strain level [13]. mNGS is highly adaptable to different sample types and nucleic acid quantities. Sample types suitable for mNGS include swabs, tissues, body fluids, and environmental specimens [7].

### 2.2. Targeted Next-Generation Sequencing (tNGS)

tNGS uses multiplex PCR amplification or probe capture to selectively enrich target microorganisms in patient samples with high sensitivity [14]. A key advantage of targeted approaches is the increased quantity and proportion of pathogen reads within sequencing data [13]. Because targeted approaches rely on primers that bind to conserved regions, they may fail to distinguish between closely related bacterial species and can completely miss viruses. The need for a predefined hypothesis and specific reagents represents a major limitation of targeted approaches [15]. While this approach limits the spectrum of detectable infections, it can enhance detection sensitivity for the targeted microorganisms. Enriching clinical samples to characterize antibiotic resistance may be a promising application of this methodology [13]. Due to its effectiveness and practicality, tNGS is increasingly used in clinical diagnostics [14].

### 2.3. Whole-Genome Sequencing (WGS)

WGS analyzes the entire—or nearly entire—genome of an organism [16]. On an individual scale, WGS enables strain typing, plasmid analysis, and the identification of resistance genes and virulence factors using a single method. At the population level, WGS can be used to characterize strains and trace evolutionary relationships among microbes during healthcare-associated or community outbreaks. In addition, WGS data also support real-time, global monitoring of strain-level epidemiology, including surveillance of resistance spread and the emergence of pathogenic mutations in humans or the environment [17]. WGS has gained increasing importance in clinical research. In particular, it is increasingly used to distinguish exogenous reinfection from relapse of the original infection—an essential step in evaluating the efficacy of treatments under investigation [16].

### 2.4. RNA Sequencing (RNA-Seq, Metatranscriptomics)

NGS can also be applied to the transcriptome—the full set of RNA transcripts (mRNA, rRNA, tRNA, microRNA, and non-coding RNA) expressed in a given cell type [11]. The indirect sequencing of RNA—following conversion to complementary DNA (cDNA)—is commonly known as RNA sequencing. cDNA RNA-seq offers a unique perspective on active biological processes. By capturing transient RNA molecules, it reflects ongoing gene activity in both host and pathogen. Detection of RNA may indicate the presence of RNA genomes or ongoing transcriptional activity within the host. Because RNA reflects a ‘snapshot’ of gene expression, transcribed RNA molecules are generally more reliable indicators of biological activity than DNA. Since DNA is more durable in the environment, DNA metagenomics examination may reveal traces of previous infections that are no longer pertinent to the diagnosis of the patient’s current state of health. The intrinsic amplification during transcription results in high RNA copy numbers, facilitating detection. Moreover, compared to DNA, RNA contains fewer repetitive and non-coding regions and conveys comparatively more functional information [15].

## 3. Materials and Methods

### 3.1. Study Design

**Figure 1:**
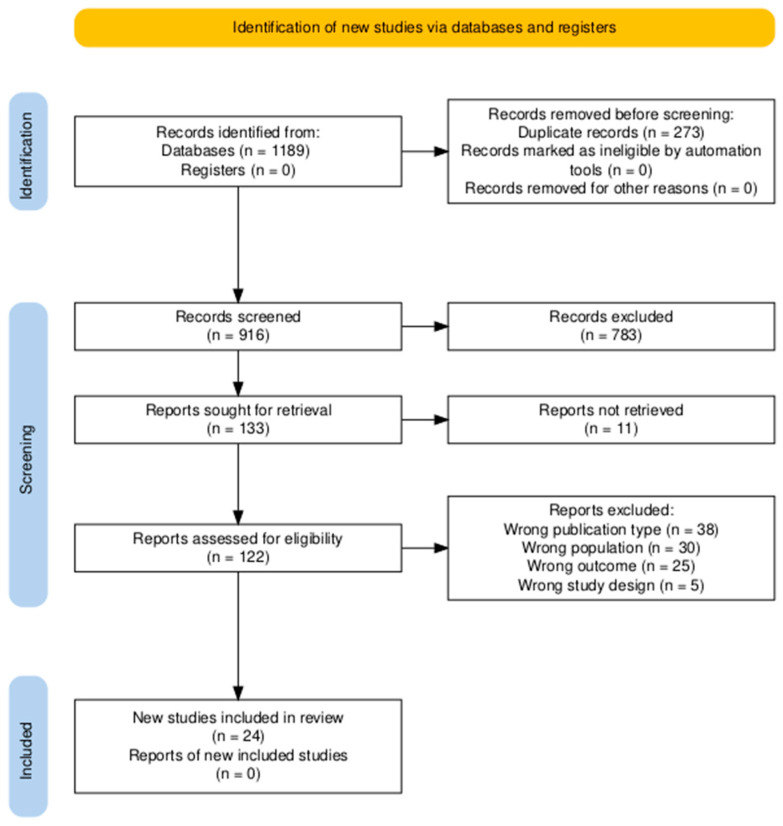
PRISMA 2020 flow diagram illustrating the process of study selection for the systematic review, including the number of records identified, screened, assessed for eligibility, and included in the final analysis [20]. A total of 1189 records were identified; after the removal of 273 duplicates, 916 were screened. Following title and abstract review, 133 full texts were assessed, and 24 studies were included.

This systematic review was conducted following the Preferred Reporting Items for Systematic Reviews and Meta-Analyses (PRISMA) 2020 statement, with relevant elements incorporated from the PRISMA-Diagnostic Test Accuracy extension for diagnostic test accuracy reviews. A completed PRISMA checklist is provided in the Supplementary Materials (Table S1), and the PRISMA flow diagram is shown in Figure 1. The review aimed to evaluate the use of next-generation sequencing technologies for diagnosing infectious diseases in pediatric oncology patients. The review protocol was developed a priori, adhering to predefined criteria for study selection, data extraction, and analysis, but was not registered in a public repository. The protocol is available from the corresponding author upon reasonable request. To enhance transparency, the full protocol is provided in Supplementary File S1 [18].

### 3.2. Eligibility Criteria

Studies were included based on the following criteria:

- Population: Pediatric patients (aged 0–18 years) with oncological diseases, including those undergoing hematopoietic cell transplantation (HCT), both autologous and allogeneic.
- Intervention: Use of NGS technologies (e.g., metagenomic NGS, targeted NGS, whole-genome sequencing, 16S/18S/ITS rRNA sequencing) for the diagnosis of infections.
- Outcomes: Diagnostic performance of NGS (e.g., pathogen identification, diagnostic yield, turnaround time), clinical relevance (e.g., impact on antimicrobial therapy), and/or patient outcomes.
- Study type: Original studies including prospective and retrospective cohorts, case series, or clinical trials.
- Language: Articles published in English.
- Timeframe: Studies published between January 2010 and April 2025.

Exclusion criteria included:

- Studies exclusively involving adult populations or lacking separate pediatric analysis.
- Focusing solely on tumor genomics or microbiome composition without an infectious disease context.
- Reviews, editorials, conference abstracts, and commentaries.

### 3.3. Information Sources and Search Strategy

A comprehensive literature search was conducted in PubMed/MEDLINE, Embase, and Scopus. The complete search strategies are presented in Supplementary Table S2. Searches were limited to articles published between 1 January 2010 and 22 April 2025, in English, involving human participants aged 0–18 years. Filters were applied to include only original research (cohort studies, case series, clinical trials) and to exclude reviews, conference abstracts, and editorials. The final search was performed on 22 April 2025. Two independent reviewers screened titles and abstracts, followed by full-text review to assess eligibility. No additional searches of reference lists, citation tracking in Google Scholar or Web of Science, or grey literature sources were conducted.

### 3.4. Study Selection

All search results were imported into Rayyan [19], a web-based tool for systematic review screening. A total of 1189 records were identified through database searching. After automatic and manual deduplication (*n* = 273), 916 records were screened based on title and abstract by two independent reviewers. During this phase, 783 records were excluded, most commonly due to wrong outcomes (*n* = 380), wrong study design (*n* = 293), or wrong population (*n* = 212). Other reasons for exclusion included wrong publication type (*n* = 46), wrong study duration (*n* = 6), foreign language (*n* = 5), and background articles (*n* = 3). Some records were excluded for multiple reasons. Of the remaining 133 articles, eleven reports could not be retrieved in full. The 122 retrieved full-text articles were then assessed against the predefined inclusion and exclusion criteria. Of these, 105 were excluded, mainly due to wrong publication type (*n* = 38), wrong population (*n* = 30), or wrong outcomes (*n* = 25). Two reviewers (A.J., A.S.) independently screened records, assessed full-texts, and extracted data; discrepancies were resolved by consensus or by a third reviewer (J.S.). Ultimately, 24 studies met the inclusion criteria and were included in the review (Figure 1).

### 3.5. Data Extraction

A standardized data extraction form was developed based on predefined review objectives. The following data were extracted from each included study:

- Basic study information (author, year, study type);
- Patient population (e.g., pediatric cancer patients, HCT recipients);
- Type of clinical sample used for NGS (e.g., blood, bronchoalveolar lavage (BAL), cerebrospinal fluid (CSF) BAL, CSF);
- NGS approach (e.g., mNGS, WGS, 16S/18S sequencing);
- Type of infection investigated (e.g., bacterial, viral, fungal);
- Pathogens detected;
- Diagnostic performance (e.g., sensitivity, specificity, positivity rate);
- Turnaround time for NGS results;
- Comparison with conventional diagnostic methods;
- Clinical impact (e.g., changes in antimicrobial treatment, diagnosis confirmation);
- Detection of antimicrobial resistance (if reported);
- Reported limitations of NGS use;
- Additional relevant notes (e.g., cost, feasibility).

Data were extracted independently by two reviewers (A.J., A.S.) and cross-checked for consistency and accuracy.

### 3.6. Risk of Bias Assessment

The quality of included studies was assessed using the Joanna Briggs Institute (JBI) Critical Appraisal Tools for case series and cohort studies. Two reviewers (A.J., A.S.) independently assessed each study for potential biases, including selection, diagnostic, and reporting bias. Any disagreements were resolved through discussion and consensus or by a third reviewer (J.S.). While QUADAS-2 is the standard tool for evaluating diagnostic accuracy studies, JBI tools were chosen here to ensure consistent assessment across the heterogeneous study designs included in this review.

### 3.7. Data Synthesis

Given the anticipated heterogeneity in study designs, NGS methods, and clinical endpoints, a narrative synthesis was conducted. Where applicable, quantitative data were summarized in tables. No transformations or imputations of data were performed; all results are presented as reported in the original studies. Missing information was not estimated. Potential sources of heterogeneity (e.g., patient population, sample type, NGS methodology, study design, and overall risk of bias) were explored qualitatively in the narrative synthesis. Due to heterogeneity in outcome reporting and study populations, meta-analysis was not conducted. This approach was chosen to enable the integration of findings from diverse study designs and NGS methodologies, where statistical pooling was inappropriate. No formal sensitivity analyses or certainty-of-evidence assessments (e.g., GRADE) were performed due to the narrative nature of the synthesis and the heterogeneity of study designs and outcomes; however, study quality and risk of bias were considered when interpreting the findings. Owing to heterogeneity in outcome reporting and study populations, formal meta-analysis was not feasible. Findings were synthesized narratively, with additional limited quantitative analyses (e.g., median yields by specimen type, ranges of management impact) to enhance interpretability across diverse study designs and NGS methodologies. We performed a simplified GRADE appraisal for four key outcomes: diagnostic yield, impact on antimicrobial management, turnaround time, and patient-centered outcomes. The certainty of evidence was rated using standard GRADE domains (risk of bias, inconsistency, indirectness, imprecision, publication bias). The summary is presented in Supplementary Table S3.

## 4. Results

This systematic review included 24 studies examining various applications of next-generation sequencing in pediatric oncology and hematopoietic cell transplantation. The included studies comprised prospective pilot trials, retrospective cohorts, and case series. The study selection process is summarized in Figure 1. In total, 1189 records were identified, and 916 remained after deduplication. Most exclusions concerned studies on tumor genomics, microbiome composition, or adult-only populations, as well as papers without infectious disease outcomes. Of the 133 full texts assessed, the majority were reviews, methodological reports, or otherwise outside the scope. Ultimately, 24 studies fulfilled the inclusion criteria. The included studies comprised prospective pilot trials, retrospective cohort studies, and case series, reflecting both exploratory and clinically implemented uses of NGS technologies. Data from all included studies were compiled into a comprehensive summary table to facilitate comparison of study characteristics, methodologies, and key findings (Table 1). Data from all included studies were compiled into four summary tables according to NGS modality (mNGS, WGS, targeted NGS, and RNA-seq) to facilitate comparison of study characteristics, methodologies, and key findings (Table 1, Table 2, Table 3 and Table 4). The full comprehensive version is provided in Supplementary Table S4.

### 4.1. Characteristics of Included Studies

This systematic review included 24 studies published between 2019 and 2025 that applied NGS technologies to the diagnosis of infections in pediatric patients with malignancies and/or after HCT. The included studies varied in design, encompassing retrospective and prospective cohorts, pilot trials, and case series, and involved both febrile neutropenic episodes and suspected infections in diverse clinical settings.

Patient populations primarily consisted of children undergoing chemotherapy for hematologic malignancies or solid tumors, as well as those after allo- or haplo-HCT. The most commonly analyzed sample types were blood or plasma (used in 18 of 24 studies, either alone or alongside other specimens), followed by bronchoalveolar lavage fluid (BALF), cerebrospinal fluid (CSF), urine, stool, nasopharyngeal swabs, and tissue specimens.

Metagenomic sequencing was the most frequently used NGS approach (*n* = 16), with other studies utilizing WGS, targeted sequencing platforms (e.g., ViroCap), 16S rRNA sequencing, and RNA sequencing, typically used for host transcriptomic profiling. A broad range of infections was investigated, including bloodstream infections, pneumonia, CNS infections, gastrointestinal and urinary tract infections, as well as unexplained fever syndromes. Across studies, frequently detected pathogens included *P. aeruginosa*, *K. pneumoniae*, *Aspergillus* spp., *Candida* spp., *Cytomegalovirus* (CMV), *Epstein–Barr virus* (EBV), adenovirus, and SARS-CoV-2. Many studies reported polymicrobial infections.

Across the 24 reports, nearly 2700 children (range 5–1025; median ≈ 65) were enrolled. Studies originated predominantly from Asia (15 reports), with additional contributions from North America (5), Europe (1), Australia (1), Africa (1), and South America (1). Roughly 70% of investigations were retrospective, four were prospective or active-surveillance cohorts [21,22,39,42], and four were designed as case series or case–control studies [34,35,37,38]. Reported median ages clustered around 6–10 years, and in >60% of studies, most participants had acute leukemia. At least three papers concentrated on the high-risk early post-HCT window (<100 days), when viral and mold infections peak [21,30,44].

### 4.2. Comparison of Diagnostic Performance Between NGS and Conventional Methods

All 24 studies reported diagnostic performance metrics for NGS. The diagnostic yield varied depending on the population, sample type, and platform. NGS demonstrated particularly high sensitivity when combined with confirmatory methods such as targeted PCR or antigen detection. For example, Shen et al. [22] reported a detection rate of 84.6% using mNGS, compared to only 7.1% with routine methods. Wang et al. [26] found that mNGS achieved a detection rate of 87.3% versus 34.5% for conventional tests in BALF samples. Other studies highlighted the ability of mNGS to detect rare, fastidious, or unexpected pathogens, including in cases of negative cultures [24,25,28].

However, sensitivity was reduced in some settings with low pathogen burden, among heavily pretreated patients, or with delays in sample processing [10]. Specificity was occasionally lower than with culture-based diagnostics, particularly for distinguishing colonization from true infection in polymicrobial contexts [29].

Across studies that reported subgroup analyses, diagnostic performance varied markedly by specimen type. BALF consistently provided the highest diagnostic yield, with positivity rates ranging from 87.3% to 100% (median ~94%). In comparison, plasma or whole blood samples achieved median yields of 81.5% (range 17.5–94.4%), while CSF showed greater variability (54.5–90.9%, median 83.3%). By contrast, conventional microbiological methods yielded median positivity rates of 28% for BALF (range 19–35%), 31% for blood (range 18–56%), and 20% for CSF (range 11–36%). These findings confirm that respiratory samples provided the highest incremental benefit for mNGS over standard diagnostics.

Mixed infections were more frequently identified by mNGS than by conventional tests, accounting for 26–60% of positive specimens, particularly in BALF and plasma cohorts. When stratified by sequencing technology, unbiased mNGS outperformed targeted platforms in overall sensitivity, whereas targeted enrichment (e.g., ViroCap, TE-NGS) demonstrated superior viral depth and recovery of novel variants.

In cohorts with febrile neutropenia or suspected bloodstream infections, mNGS performed on plasma or blood identified pathogens in 69–86% of episodes, substantially outperforming culture or targeted PCR, which detected pathogens in only 18–56% of cases—representing a two- to fivefold increase in diagnostic yield [21,40]. Sensitivity for culture-confirmed bacteremia reached 89–96% across three independent series [28,32,33].

In pulmonary disease, mNGS of bronchoalveolar lavage fluid achieved high positivity rates up to 90% in one pediatric cohort [27] and substantial added value in another study [44].

In neutropenic children, mNGS more often revealed bacterial and fungal pathogens, whereas in non-neutropenic patients, viral reads predominated [26,33]. For example, Wu et al. [33] reported significantly higher incidence of bacterial bloodstream infection in neutropenic patients (43.5% vs. 20.0%) and lower frequency of viral infections (52.2% vs. 76.7%). By contrast, in cohorts with ANC ≥ 0.5 × 10^9^/L, mNGS frequently identified respiratory viruses, sometimes of uncertain clinical relevance [26].

One bloodstream-sepsis study reported an AUC of 0.89 for mNGS versus 0.54 for blood culture [33]. Adding the host biomarker interleukin-6 ≥ 390 pg/mL increased mNGS specificity from 71% to 86% without compromising sensitivity [29].

### 4.3. Influence on Clinical Management

NGS results influenced clinical decision-making in most studies. mNGS findings led to antimicrobial therapy modifications, including escalation, de-escalation, or initiation of targeted antifungal treatment [32,40]. In a study by Wang et al. [26], therapy was adjusted in over 85% of patients with pulmonary infections based on mNGS results.

Early application of mNGS facilitated etiologic clarification and supported the de-escalation of broad-spectrum therapy, especially in febrile neutropenic patients with negative conventional tests [23,31]. In studies by Xu et al. [32] and Fu et al. [28], mNGS was associated with shorter fever duration, reduced antimicrobial use, and lower hospitalization costs.

Nevertheless, the clinical utility of NGS was limited in some studies due to long turnaround times, the uncertain clinical relevance of the detected organisms, or a lack of real-time availability [10,34]. In the study by Jalal et al. [25], WGS was mainly used for resistance profiling and outbreak tracking, without direct impact on individual therapy.

Among studies reporting such data, therapy was modified in a median of 63% of evaluable episodes (range 13–96%), most often through escalation or the addition of targeted antifungals after mNGS results [27,28,44].

Across studies that reported antimicrobial management outcomes (*n* = 18), mNGS results led to changes in therapy in a median of 63% of evaluable episodes (interquartile range 42–85%, range 13–96%). Escalation or targeted initiation of antifungal or antiviral therapy was the most frequent intervention, occurring in 38% (range 25–67%) of modified cases, while the de-escalation or discontinuation of broad-spectrum antibiotics occurred in 27% (range 13–40%).

When quantified across reports, the median rate of therapy modification attributable to mNGS was 54% (range 13–92%), with the highest impact observed in BALF-derived testing (61–88% of patients). Blood- or plasma-based assays influenced management in approximately half of evaluable episodes (27–54%), most often through antimicrobial escalation or the addition of targeted antifungals or antivirals. Mortality outcomes also reflected the added value of sequencing; in one PICU cohort, mortality reached 42.9% among mNGS-negative patients compared with 7.1% in those with positive actionable results [29].

The timing of sequencing emerged as a critical determinant of clinical utility. Fu et al. demonstrated that children tested within 48 h of symptom onset had significantly shorter fever duration (median 4.9 vs. 11.6 days) and lower antimicrobial as well as total hospitalization costs compared to those tested later [28]. Similarly, early mNGS application was associated with earlier antimicrobial adjustment and improved survival in intensive care settings [29]. These findings underscore that prompt sequencing maximizes clinical impact.

The de-escalation or discontinuation of broad-spectrum cover was implemented in 25–40% of febrile neutropenia episodes. Two studies that implemented early (<48 h) mNGS documented a 1.5-day reduction in fever duration and 25–30% lower total antimicrobial costs [28,32].

Beyond bedside care, a prospective WGS-based surveillance program uncovered 18 silent transmission clusters that routine epidemiology had missed, prompting focused infection-control interventions [39]. Conversely, two large clinical series applying plasma or BALF mNGS reported turnaround times exceeding 7 days and actionable findings in only ≈13% of test requests [10,34].

### 4.4. NGS Methodologies and Sample Types

mNGS was the dominant sequencing approach (16 of 24 studies). Targeted sequencing platforms like ViroCap were employed for viral diagnostics [40], while 16S rRNA gene sequencing supported bacterial identification in cases of sinusitis [43]. WGS was used for antimicrobial resistance profiling and epidemiological surveillance [36,37,38]. RNA-seq was applied in one study to analyze the host immune response and distinguish infectious from non-infectious fevers, achieving >85% accuracy and suggesting that up to one-third of febrile neutropenia episodes may have a non-infectious etiology [42]. Some studies enhanced clinical interpretation by integrating host biomarkers such as IL-6 [27].

Blood and plasma were the most frequently used sample types (*n* = 18), followed by BALF and CSF. Across the dataset, blood or plasma samples accounted for approximately 46% of all NGS tests, bronchoalveolar lavage fluid 21%, cerebrospinal fluid, urine, or stool 18%, and tissue aspirates 7%. Positivity rates for mNGS typically exceeded 75%, reaching >90% in some BALF cohorts [25,26]. Mixed bacterial–fungal–viral infections were common, occurring in up to 50% of BALF specimens and ≈35% of blood samples, underscoring the value of unbiased sequencing in these highly immunocompromised hosts [29,32].

Several technological variants extended the reach of NGS beyond standard DNA metagenomics. Cell-free DNA sequencing allowed fully non-invasive detection of deep-seated mold disease in high-risk oncology and transplant patients [21]. Target-enrichment workflows such as ViroCap and TE-mNGS increased viral read depth by one to two orders of magnitude, enabling recovery of complete genomes of human pegivirus-1 and a novel astrovirus that routine PCR had missed [40,41]. For culture isolates, rapid whole-genome sequencing of multidrug-resistant *A. baumannii* delivered resistance profiles within six hours and underpinned successful outbreak control on a pediatric cancer ward [36].

### 4.5. Detection of Antimicrobial Resistance (AMR) and Surveillance Applications

The detection of antimicrobial resistance genes using next-generation sequencing technologies was not the primary focus in most studies included in this review, but a few reports explored its potential. Horiba et al. [23] examined resistance gene profiles in bloodstream infections, highlighting the feasibility of detecting AMR determinants directly from clinical samples. Their study underscored the potential clinical relevance of genotypic resistance screening, although the interpretation of resistance markers in polymicrobial infections remained complex. Jalal et al. [36] combined WGS and phenotypic susceptibility testing to characterize multidrug-resistant *A. baumannii* isolates, demonstrating strong concordance between genetic and phenotypic resistance data. This example illustrates how WGS can support real-time antimicrobial stewardship and genomic epidemiology, particularly in the context of highly resistant pathogens.

Nevertheless, the majority of studies did not report AMR detection or only provided limited or qualitative findings, often without systematic validation. Several authors explicitly stated that resistance analysis was not performed or not feasible due to limitations in sequencing depth, targeted panels, or annotation pipelines [21,22]. Furthermore, no studies systematically evaluated the clinical utility of AMR prediction from NGS data in terms of patient management decisions. While the integration of resistome analysis into routine metagenomic diagnostics holds promise, it remains underexplored in pediatric immunocompromised populations and requires standardized methodologies and robust phenotypic correlation studies to support clinical translation.

### 4.6. Limitations of Evidence

Despite increasing enthusiasm for sequencing-based infectious disease diagnostics, the available evidence in pediatric hematology–oncology and transplant populations remains limited and heterogeneous. The included studies showed substantial variability in sequencing platforms, sample types, patient populations, and bioinformatic pipelines. This lack of standardization hampers direct comparison and limits the generalizability of findings. Moreover, many studies were retrospective, small in scale, or based on case series, limiting the strength of inference and precluding formal assessments of diagnostic accuracy and clinical impact. The certainty of evidence was generally rated as low to very low across all outcomes, reflecting observational designs, substantial heterogeneity, and inconsistent reporting (Supplementary Table S3).

Another key limitation was the frequent lack of a clear reference standard, as many studies did not validate NGS results against conventional microbiological tests. This may have led to misclassification and potential overestimation of diagnostic performance. Recurrent technical challenges included high sequencing costs, prolonged turnaround times, and limited access to comprehensive reference databases. In polymicrobial or low-biomass specimens, distinguishing true pathogens from contaminants or commensals was frequently problematic. As Horiba et al. [23] noted, attributing AMR genes to specific organisms in such contexts remains complex, underscoring the bioinformatic challenges inherent to metagenomic data interpretation.

Furthermore, few studies assessed clinical endpoints such as therapy modification, length of hospitalization, or outcomes attributable to NGS-guided diagnosis. Moreover, even when clinical endpoints were reported, heterogeneity in definitions of impact and inconsistent stratification by specimen type (blood, BALF, CSF) limited comparability, underscoring the need for standardized outcome reporting in future prospective studies. When performed, AMR profiling was often restricted to the detection of select genes and was rarely incorporated into actionable clinical frameworks. The paucity of prospective, adequately powered studies underscores the need for future research to address these gaps and rigorously evaluate the cost-effectiveness, feasibility, and clinical impact of NGS implementation in real-world pediatric oncology and transplant settings.

Risk of bias was assessed using the Joanna Briggs Institute (JBI) appraisal tools. Overall methodological quality was variable. Only one study was rated as low-to-moderate risk [39], two were assessed as high risk [10,36], while the remaining studies were classified as either high-to-moderate (*n* = 11) or moderate risk (*n* = 10). Common limitations included retrospective design, small sample sizes, a lack of blinding, and the absence of a reference standard. In several studies, the interpretation of mNGS results was subjective or lacked validation against conventional diagnostics. Analyses of clinical impact were often limited or descriptive. A detailed study-level summary (author, year, study design, overall JBI rating, and main sources of bias) is presented in Supplementary Tables S3 and S5.

## 5. Discussion

This review evaluated the clinical utility, diagnostic performance, and implementation of NGS for detecting infectious pathogens in pediatric patients with malignancy and/or after hematopoietic cell transplantation. Given the high risk of severe infections in these immunocompromised populations, early and accurate pathogen identification is critical to improving outcomes and guiding targeted therapy.

Across 24 heterogeneous studies, NGS often outperformed conventional diagnostics, especially for viral and fungal infections, and yielded clinically actionable results in most cases. In several studies, NGS findings directly influenced antimicrobial decision-making by enabling pathogen-directed treatment or helping avoid unnecessary therapy in cases of non-infectious fever.

These findings support growing evidence that NGS—particularly unbiased metagenomic approaches—can serve as a valuable adjunct to traditional diagnostics when conventional methods are inconclusive. However, the interpretation of NGS data remains complex, and the absence of standardization across laboratories and studies continues to limit broad clinical implementation. Moreover, the real-world impact of NGS on outcomes such as mortality, length of stay, and cost-effectiveness remains to be firmly established.

### 5.1. Pathogen Detection Sequencing

An important dimension of mNGS performance is the type of pathogen identified. Across cohorts, viral reads (especially CMV, EBV, and HHV-6/7) were the most frequent, yet often clinically ambiguous, as asymptomatic shedding or latent reactivation may not represent the true etiology of fever [26,32]. In contrast, fungal and mixed infections provided the clearest added value: *P. jirovecii*, *Mucorales, Trichosporon*, and other opportunists were consistently missed by conventional tests but promptly recognized by sequencing [25,29]. Mixed infections were reported in up to 50% of BALF and one-third of plasma samples, illustrating how unbiased sequencing captures the full complexity of infectious syndromes in immunocompromised children [26,32].

Current evidence supports early plasma/whole-blood mNGS in culture-negative sepsis and persistent febrile neutropenia, and BALF mNGS in severe or atypical pneumonias [26,28,32]. To maximize clinical impact, laboratories should (1) integrate sequencing into a real-time WGS surveillance pipeline for outbreak detection [39]; (2) link results to antimicrobial stewardship frameworks capable of acting on resistome predictions; and (3) enroll in the external quality-assessment schemes now required by the 2024 ISO 15189 amendments. Implementation remains hindered by high per-test costs and fragmented regulatory frameworks. The 2024 Foundation for Innovative New Diagnostics (FIND) Diagnostic Readiness Index also highlights limited access to advanced sequencing in low- and middle-income countries [44].

Comparative evaluation of platforms suggests complementary strengths. DNA-based mNGS was most widely applied and delivered the highest sensitivity for bacterial and fungal infections [26,28]. Target-enrichment approaches, such as ViroCap or TE-NGS, achieved deeper viral coverage and enabled the discovery of unusual or emerging viruses, including human pegivirus-1 and astrovirus VA3, missed by PCR [40,41]. RNA-seq provided host transcriptomic signatures that distinguished infectious from non-infectious fever episodes with >85% accuracy [42], highlighting its potential to reduce overtreatment. Finally, isolate-based WGS was invaluable for resistance profiling and outbreak tracing, exemplified by *A. baumannii* and adenovirus transmission clusters [36,37]. These findings indicate that the optimal sequencing approach depends on the clinical context—pathogen detection, resistance assessment, or host-response profiling.

### 5.2. Host-Response, Microbiome, and Host-Genome Sequencing

Although metagenomic sequencing consistently outperformed conventional microbiology, its true value lies in how incremental detections translated into clinical care. Beyond detection rates, three practice-changing insights deserve emphasis. Firstly, timely test ordering is pivotal: performing mNGS within the first two days of fever reduces both febrile duration and antimicrobial use, highlighting its value at the start of the diagnostic pathway [31,40]. Secondly, actionability is high—nearly two-thirds of positive cases prompted escalation (typically to mold-active therapy) or de-escalation once a viral etiology was confirmed [21,30], reinforcing the role of mNGS as both a stewardship and life-saving tool. Thirdly, sequencing provides value beyond detection. For example, resistome-aware WGS enabled resolution of an *A. baumannii* outbreak within hours. [25], and ward-wide surveillance identified 18 silent transmission clusters that routine epidemiology had missed [39]. Taken together, these findings suggest that mNGS should be seen not as a last-resort assay, but as an integrated platform spanning diagnosis, antimicrobial stewardship, and infection control.

These findings mirror adult data, where metagenomic sequencing provides a significant diagnostic yield advantage over culture, while also highlighting pediatric-specific benefits. While adult series occasionally report clinically irrelevant reads or contaminants [45], the pathogen spectrum in children receiving intensive prophylaxis and chemotherapy shifts toward viruses and molds—organisms largely undetectable by routine culture—thus amplifying the value of unbiased sequencing. Nevertheless, a recent Journal of Clinical Investigation review emphasizes the persistent lack of pediatric-tailored guidelines and reiterated the same bioinformatic, regulatory, and reimbursement barriers identified in our analysis [46].

Beyond pathogen detection, NGS is beginning to transform our understanding of host–microbe interactions and genetic susceptibility, addressing complementary clinical questions. Beyond routine diagnostics, next-generation sequencing is beginning to reveal host–microbe interactions that influence infection risk in pediatric hemato-oncology. Shotgun metagenomics of stool samples has shown that intensive chemotherapy and broad-spectrum antibiotics cause profound dysbiosis. Loss of α-diversity and *Enterococcus* overgrowth are associated with complicated febrile neutropenia following hematopoietic cell transplantation [47,48]. Longitudinal sequencing in the same setting demonstrates rapid acquisition of resistance genes such as *vanA and aac(6′)-Ie-aph(2″)-Ia* soon after antibiotic exposure, suggesting that a patient-level resistome read-out could guide empiric therapy [48,49].

Analysis of the host genome by germline WES/WGS increasingly reveals variants that both predispose to malignancy and impair immune defense. For example, pathogenic or likely pathogenic alleles in *GATA2*, *ASXL1*, or *TET2* were found in 10% of pediatric chronic myeloid leukemia cases, with additional putative risk alleles in another 60% [49,50]. These data raise the prospect of targeted prophylaxis or intensified surveillance for variant carriers.

NGS is also transforming the diagnosis of inborn errors of immunity. Rapid panels or whole-exome sequencing can provide a molecular diagnosis within a week, enabling tailored antimicrobial prophylaxis and optimal timing of HCT. A recent review advocates routine screening in children with unusual or recurrent infections [49,50].

Finally, serial metagenomic ‘snapshots’ of the gut resistome can forecast bloodstream infections and track the emergence of multidrug-resistant clones before clinical symptoms appear. These insights may support the development of dynamic stewardship dashboards integrating patient-specific resistome data with local antibiograms [48,49].

### 5.3. Overall Interpretation and Limitations

Collectively, pathogen, host-genome, and microbiome sequencing illustrate how integrated approaches can advance precision infection–oncology by enabling personalized diagnostics, risk stratification, and targeted prevention. 

A recurring limitation across studies was the occurrence of false-positive and false-negative results. In the study by Lehman et al. [10], plasma mNGS showed only moderate concordance with conventional diagnostics and frequently detected incidental or non-pathogenic organisms, some of which led to negative clinical consequences, underlining the risk of false positives. Qu et al. [25] similarly noted interpretive challenges in BALF samples, where contamination, delayed processing, and the lack of RNA sequencing contributed to both false-positive and false-negative results. Conversely, Shen et al. [22] reported that although mNGS guided antimicrobial adjustment in most patients, it failed to detect clinically important pathogens such as *P. jirovecii* and *A. fumigatus* in a subset of cases, illustrating the risk of false negatives. These examples highlight the need for standardized analytical thresholds, contamination controls, and the integration of sequencing results with clinical and radiologic data to avoid misinterpretation.

While the potential of NGS is clear, important limitations temper the interpretation of our findings. The primary literature is markedly heterogeneous, with studies differing in sequencing platforms, library preparation methods, host-read depletion techniques, and taxonomic cut-offs, preventing the pooling of effect estimates in a meta-analysis. The overall certainty of evidence was judged as low to very low across key outcomes, underscoring the need for larger, prospective studies with standardized endpoints. Although risk-of-bias assessments were performed independently by two reviewers, they remain partly subjective. Moreover, most studies lacked independent confirmation, blinding, or standardized reference criteria. Key implementation metrics—such as turnaround time, cost, and patient-centered outcomes—were rarely reported in a consistent format, precluding formal cost-effectiveness or outcome modeling. Another key limitation is that most included studies did not assess patient-centered outcomes such as mortality, intensive care admission, or length of hospitalization, which precludes conclusions about the true clinical benefit of NGS-guided diagnosis. Limitations of this review include restriction to English-language publications, the use of only three bibliographic databases without grey literature searches, the lack of protocol registration, and no contact with study authors to obtain missing data, which may have increased the risk of omitting relevant studies. The protocol was not prospectively registered, as the review was initially designed as part of an academic doctoral project and primarily anticipated a narrative synthesis due to study heterogeneity. Nonetheless, the full protocol is available in Supplementary File S1 to enhance transparency. Finally, since this review focused exclusively on children with cancer or undergoing HCT, the findings should be extrapolated to other immunocompromised populations with caution.

### 5.4. Future Directions

Future research should focus on well-designed, prospective, randomized trials comparing mNGS-based therapy with current standards, including cost-effectiveness analyses in different healthcare settings. Such studies should also incorporate standardized patient-centered outcomes, including mortality, length of hospitalization, intensive care unit admission, and time to appropriate therapy, to determine whether the diagnostic advantage of NGS translates into measurable clinical benefit. The development of a uniform reporting framework—covering sample handling, contamination control, taxonomic thresholds, and phenotypic correlation—is essential to ensure the comparability and reproducibility of results, paving the way for precise, integrated approaches to the treatment of infections in pediatric oncology. These findings have direct implications for clinical practice, guiding the targeted use of NGS in pediatric patients with high-risk oncology; for policy, highlighting the need for standardization and reimbursement frameworks; and for research, emphasizing prospective studies, cost-effectiveness assessments, and pediatric-specific diagnostic guidelines.

## 6. Conclusions

Next-generation sequencing now enables pediatric oncologists to detect hard-to-culture pathogens within 24–48 h. These rapid results support timely escalation to mold-active or antiviral therapy and allow for confident de-escalation when a non-bacterial etiology is identified. Whole-genome sequencing of clinical isolates adds ward-level value by uncovering silent transmission clusters and informing targeted infection-control interventions. Although high costs and the need for laboratory standardization still limit widespread adoption, interpretation of diagnostic accuracy is further constrained by the lack of consistent reference standards across studies. Nevertheless, current evidence supports the use of mNGS in selected high-risk scenarios, including culture-negative sepsis, persistent febrile neutropenia, and severe or atypical pneumonia. When integrated into antimicrobial stewardship and surveillance frameworks, NGS transitions infection management from empiricism to data-driven care. It lays the foundation for precision infection-oncology, where clinical decisions are guided by pathogen identity, resistance profiles, and host immune risk.

## Figures and Tables

**Table 1:** Studies using metagenomic next-generation sequencing for infection diagnosis in immunocompromised pediatric patients with malignancies.

Clinical Impact	Comparison with Conventional Methods	Diagnostic Performance	Type of Infection/Pathogens Detected	NGS Approach	Sample Type	Patients Population	Study Type	Author
cfDNA NGS identified fungal pathogens in some high-risk patients but was not available in real-time and did not influence treatment decisions. Despite negative NGS results, 72% of patients without proven IFD received ≥1 week of antifungal therapy.	NGS showed good concordance with invasive fungal diagnostics (lung, pancreatic, scalp), detecting pathogens at the species level. Missed some tissue-only findings (e.g., *Rhizopus oryzae*), but identified *P. jirovecii* not found by conventional methods.	NGS identified fungal pathogens in 7 of 40 high-risk patients and matched conventional diagnostics in 4 of 6 proven IFD cases. Missed detections attributed to limited cfDNA release or uncertain clinical relevance.	Polymicrobial infections detected, including fungal (e.g., *Aspergillus fumigatus*, *Candida spp.*, *Rhizopus delemar*, *Pneumocystis jirovecii*), viral (e.g., CMV, BK virus, HHV-6A, VZV), and bacterial pathogens (e.g., *Escherichia coli*, *Pseudomonas spp.*, *Enterococcus spp.*, *Streptococcus mitis*, *Helicobacter pylori*).	cfDNA NGS	Blood samples.	40 pediatric hematology, oncology, and stem cell transplant patients at risk for invasive fungal disease (IFD).	Prospective observational cohort study	Armstrong et al., 2019 [21]
mNGS guided antimicrobial adjustment in 55/70 (78.6%) patients, leading to clinical improvement. In 21.4%, results were irrelevant or missed likely pathogens (e.g., *P. jirovecii*, *A. fumigatus*), highlighting both utility and limitations in real-world use.	mNGS detected all pathogens found by conventional tests (5/70; 7.1%) and identified additional clinically relevant microbes in cases with negative routine diagnostics. Enabled overlapping detection across sample types (e.g., blood + swab in 14/34 cases).	mNGS showed a high detection rate (84.6%; 88/104 samples), identifying pathogens in both plasma and respiratory samples, including polymicrobial and rare infections. Enabled pathogen detection even when routine tests were negative.	Bacterial, viral, and fungal pathogens detected by mNGS, including *Pseudomonas aeruginosa*, *Klebsiella pneumoniae*, *Staphylococcus aureus*, *Candida albicans*, EBV, CMV, HSV-1, HHV-7, *parvovirus B19*, adenovirus, rhinovirus, and polyomaviruses.	mNGS	Plasma (*n* = 62), throat swabs (*n* = 34), bone marrow (*n* = 4), bronchoalveolar fluid (BALF) (*n* = 4); total 104 samples.	70 febrile pediatric patients with hematological disorders; mostly immunocompromised due to chemotherapy, HCT, or underlying disease.	Prospective observational diagnostic study	Shen et al., 2021 [22]
NGS findings suggest that translocation of oral, skin, and gut microbiota may contribute to FN pathogenesis in neutropenic patients. Potential to reveal hidden sources of infection when cultures are negative.	NGS confirmed all culture-positive FN cases and identified additional pathogens in culture-negative cases, demonstrating complementary value to conventional diagnostics.	NGS detected pathogens in 5/10 FN patients with positive cultures, 15/87 (17%) culture-negative FN cases, and 3/8 neutropenic enterocolitis patients. Showed added value in cases with negative standard diagnostics.	Putative bacterial pathogens detected in 15/87 culture-negative FN cases; confirmed all 5 culture-positive FN cases. DNA viruses (e.g., CMV, HHV-6B, EBV, TTV) in 19 patients; *Malassezia restricta* in 1 case. Findings suggest flora translocation.	mNGS	Plasma/serum samples.	Plasma/serum samples of 112 pediatric patients with FN and 10 patients with neutropenia without fever.	Retrospective diagnostic cohort study	Horiba et al., 2021 [23]
mNGS revealed a high rate of co-infections (35.7%), emphasizing the need for careful clinical correlation. Frequent detection of viruses not matching symptoms highlighted the importance of integrating mNGS with host-response markers and clinical judgment.	Outperformed conventional methods in FUO by detecting pathogens in cases missed by standard tests; added microbiological value in 27.9% of patients.	mNGS identified pathogens in 76.2% (112/147) of FUO cases with prior negative conventional tests. Pathogens were considered causative in 44.6%, and clinical resolution followed therapy adjustment in 27.9%.	Bacterial, viral, and fungal pathogens detected: most common were *P. aeruginosa*, *K. pneumoniae*, *Acinetobacter baumannii*, CMV, HHV-1, *parvovirus B19*, *A. fumigatus*, and *Candida parapsilosis*.	mNGS	Blood samples.	147 pediatric patients with hematological malignancies undergoing fever of unknown origin (FUO) (after chemotherapy or HCT).	Retrospective observational diagnostic study	Zhang et al., 2022 [24]
mNGS supported antibiotic guidance through faster and broader pathogen detection, despite false negatives/positives. Interpretation was challenged by sample type (e.g., BALF contamination), delayed processing, and absence of RNA sequencing, limiting viral detection.	Compared to culture and RT-PCR, mNGS detected significantly more pathogens, especially fungi and viruses. mNGS outperformed conventional methods in BALF, blood, and CSF samples in terms of sensitivity, and enabled detection of mixed and atypical infections.	Higher sensitivity of mNGS vs. conventional tests (89.7% vs. 21.8%), especially for fungal and viral infections; slightly lower specificity (78.5% vs. 92.9%).	mNGS identified pathogens in 91.7% BAL, 85.7% blood, and 73.3% CSF samples; common pathogens: CMV, *P. jirovecii*, *P. aeruginosa*, *K. pneumoniae*, HHV-6B, *Aspergillus*, *Mucor*; mixed infections in 6 BAL and 5 blood cases.	mNGS	BALF (*n* = 54), blood (*n* = 32), and cerebrospinal fluid (CSF) (*n* = 15) samples.	101 pediatric recipients after allo-HCT.	Retrospective observational diagnostic study	Qu et al., 2022 [25]
mNGS supported timely and targeted antibiotic adjustments in cases not covered by empirical therapy, potentially improving outcomes. It also enabled de-escalation in selected cases. Authors recommend early BAL mNGS in children with hematologic malignancy and pulmonary infection.	Conventional tests included culture, serology (e.g., RSV, CMV, EBV), and fungal antigen assays (GM/G-test). mNGS detected mixed infections in 30.9% vs. 7.3% by conventional methods, especially bacterial–viral and fungal–viral co-infections.	mNGS alone had a higher positivity rate (87.3%) than conventional methods (34.5%, *p* < 0.001); it detected bacteria in 31%, viruses in 45.5%, and fungi in 34.5%. Mixed infections identified in 31% of cases. Combined with conventional tests, etiology was established in 91%.	Pulmonary infections with bacterial (e.g., *Streptococcus pneumoniae*, *Haemophilus influenzae*, *S. aureus*), viral (e.g., CMV, RSV, HPIV3, EBV), and fungal pathogens (e.g., *P. jirovecii*, *A. fumigatus*, *R. oryzae*) detected by mNGS in BALF.	mNGS	BALF samples.	55 children with hematologic malignancies and suspected pulmonary infections undergoing bronchoscopy.	Retrospective cohort study	Wang et al., 2022 [26]
mNGS improved etiologic diagnosis in FUO cases and enabled more targeted antimicrobial decisions. Integration with IL-6 enhanced clinical interpretation of ambiguous or mixed results, supporting precision treatment in immunocompromised children.	mNGS detected pathogens in 59.7% of cases missed by conventional methods, while only 3.1% were positive by conventional tests alone. Agreement was limited (27.1%), with differing results in 37% of concordant positives. mNGS had higher positivity rates across all sample types, especially NPS (94.3% vs. 18.9%) and BALF (96.7% vs. 36.7%).	mNGS was positive in 86.8% of cases vs. 30.2% with conventional methods; 59.7% of infections were detected by mNGS only. In 71.4% of mNGS-positive cases, pathogens were considered clinically relevant. IL-6 ≥ 390 pg/mL improved diagnostic precision for bacterial infections in mixed results.	mNGS detected bacteria (e.g., *Haemophilus parainfluenzae*, *P. aeruginosa*, *K. pneumoniae*), viruses (e.g., CMV, EBV, HHV-7, HSV-1, parvovirus B19), and fungi (e.g., *P. jirovecii i*, *A. fumigatus*, *C. parapsilosis*, *Fusarium spp.*). Co-infections (bacteria/viruses/fungi) were frequent; >50% of positives involved ≥2 pathogens.	mNGS	Blood (*n* = 157), nasopharyngeal swabs (*n* = 53), BALF (*n* = 30), sputum (*n* = 6), pus (*n* = 5), hydrothorax/ascites (*n* = 4), CSF (*n* = 3).	258 febrile pediatric patients with leukemia, lymphoma, other malignancies, or HCT; infections included RTI/pneumonia, FUO, bloodstream infection (BSI), abdominal, CNS, GI, oral, and soft tissue infections.	Retrospective observational study	Wang et al., 2022 [27]
Early use of mNGS (<48 h) in febrile, immunocompromised children was associated with shorter fever duration, lower anti-infective and hospitalization costs. High sensitivity in myelosuppressed patients enabled faster etiologic diagnosis and better-targeted treatment.	mNGS yielded significantly higher positivity than conventional methods (83.3% vs. 17.7%, *p* < 0.05); detected pathogens in 80 vs. 17 events, respectively.	mNGS showed higher sensitivity (91.8%) and NPV (56.3%) than conventional methods (17.7% and 11.4%, respectively), with similar specificity (81.8%). PPV was 97.5% for mNGS vs. 88.2% for conventional testing.	Detected bloodstream infections (76%), pneumonia (44.8%), and UTI (2.1%). Most common pathogens: *P. aeruginosa* (20.5%), *K. pneumoniae* (8.7%), CMV (21.3%), and *Candida spp*. (12.6%).	DNA-based mNGS (Illumina platform)	Blood, CSF, BALF, sputum, urine, and tissue samples; total *n =* 127 (including 107 blood, 6 CSF, 2 BALF, 3 sputum, 7 urine, 2 tissue).	70 febrile pediatric patients (median age 5 y) with malignancies or hematologic disorders, including ALL, AML, NHL, LCH, aplastic anemia, RB, and Evans syndrome; all underwent mNGS testing.	Retrospective observational study	Fu et al., 2022 [28]
mNGS results influenced clinical management in ~35% of cases, leading to initiation of targeted therapy in 39 patients and de-escalation in 3. Findings support mNGS as a complementary tool, particularly in culture-negative FN, though interpretation requires caution.	Outperformed conventional methods in detection rate (63.2% vs. 42.5%, *p* < 0.001); better at identifying mixed and rare infections.	mNGS showed higher sensitivity (90.9%) but lower specificity (12%) vs. TPD (sensitivity 24.7%, specificity 100%). mNGS positivity rate was 85.7% vs. 38.8% for TPD (*p* = 0.000); AUC for mNGS was 48.5%.	mNGS detected 70 pathogenic strains in 42 FN cases, including 25 mixed infections. Predominant pathogens included *Aspergillus spp.* and Gram-negative bacteria. *Aspergillus* was detected in 19 cases (often G-test/GM-test negative), and 13/20 Gram-negative bacteria were culture-negative but mNGS-positive.	mNGS	Plasma (*n* = 49) and BALF (*n* = 12).	49 children with febrile neutropenia after chemotherapy; majority with leukemia (ALL: 65.3%, AML: 30.6%); 2 cases with HLH.	Retrospective cohort study	Guo et al., 2022 [29]
Early HAdV detection by mNGS may enable timely antiviral treatment and improve outcomes. Highlights the value of mNGS in diagnosing adenovirus infections in immunocompromised pediatric patients post-haplo-HCT.	mNGS offers a comprehensive diagnostic approach and may detect infections missed by conventional methods; no direct comparison or statistical analysis reported.	mNGS successfully identified HAdV infections post-haplo-HCT; no formal sensitivity or specificity metrics reported.	Systemic human adenovirus (HAdV) infection involving blood, urine, CSF, and lungs; clinical manifestations included ADV hepatitis and encephalitis.	mNGS	Blood, CSF, urine, and BALF samples.	7 patients with adenovirus infection after haploidentical HCT (from cohort of 976); 6 with acute leukemia, 1 with aplastic anemia; 5 male, 2 female.	Retrospective multicenter study	Wu et al., 2023 [30]
mNGS guided treatment adjustments (e.g., linezolid initiation, antifungal addition, antibiotic de-escalation), with most patients improving clinically. Enabled early differentiation between infectious and non-infectious fevers.	mNGS showed higher and earlier pathogen detection than culture, identifying organisms missed by conventional methods. In one case, *K. pneumoniae* was confirmed by culture three days after mNGS detection.	mNGS showed higher positivity for bacteria and fungi (57.2%) compared to culture (12.5%, *p* < 0.01); detected bacteria (*n* = 27), fungi (*n* = 12), viruses (*n* = 7), and mixed infections (*n* = 16). No fungi were detected by culture.	Infections included sepsis, respiratory tract infections, and febrile neutropenia with suspected bloodstream infection. mNGS identified pathogens in 85/96 specimens (88.5%), including simple bacterial (43.5%), fungal (19.4%), viral (11.3%) and mixed infections (25.8%). Frequent pathogens: *K. pneumoniae*, *P. aeruginosa*, *A. baumannii*, *E. coli*, *Yersinia pneumoniae*, *Aspergillus flavus*, and human herpesviruses.	mNGS	96 specimens: plasma (*n* = 71), CSF (*n* = 11), sputum (*n* = 8), BALF (*n* = 2), hydrothorax, urine, liver biopsy, and abscess fluid (*n* = 1 each).	67 pediatric patients with hematological diseases, mostly ALL (52.2%) and AML (14.9%); 28.4% post-HCT; 39 males.	Retrospective observational study	Zhang et al., 2023 [31]
mNGS influenced clinical diagnosis in 91.7% and led to treatment modifications in 95.8% of immunocompromised patients. The study highlights the value of mNGS for rapid, comprehensive pathogen detection in critically ill immunocompromised children, where infections may be atypical or otherwise undiagnosed.	mNGS showed superior pathogen detection compared to conventional microbiological testing (CMT), with a significantly higher positivity rate (76.5% vs. 55.5%). mNGS identified additional pathogens missed by CMT, especially in mixed infections and among immunocompromised patients. In some cases, mNGS provided the only microbiologic evidence of infection.	mNGS positivity rate: 76.5%, significantly higher than conventional testing (55.5%, *p* = 0.0006). Positive percent agreement was higher in immunocompromised patients (95.2%) vs. immunocompetent (77.8%).	Detected Gram-positive and Gram-negative bacteria (e.g., *S. pneumoniae*, *K. pneumoniae*), Herpesviridae viruses (CMV, EBV, HSV), respiratory viruses (RSV, HPIV, HRV), and fungi including *P. jirovecii* and *C.albicans*. Co-infections were common in immunocompromised children.	DNA mNGS (BGI platform)	119 clinical samples, including BALF, CSF, blood, stool, peritoneal fluid, pleural fluid, pus, sputum, and swabs; 48 samples from immunocompromised patients.	48 immunocompromised pediatric patients (avg. age 88 mo), mainly with leukemia (52.1%) and solid tumors (20.8%). Most common symptoms: fever (84.9%), cough (29.4%), convulsions (2.1%). Total of 119 samples analyzed.	Retrospective cohort study	Xu et al., 2024 [32]
mNGS guided treatment changes (54.3%), improved diagnostic confidence, reduced unnecessary antibiotics, and enabled precise therapy in pediatric cancer patients with suspected BSI.	mNGS significantly outperformed conventional microbiological tests (culture, PCR for EBV/CMV, GM/G tests) in sensitivity, clinical agreement, and pathogen coverage—especially for viruses. While both methods were positive in 25.4% of samples, only 15.8% showed identical pathogen identification. mNGS detected 94.5% of clinician-confirmed pathogens, including 100% of viruses, and was the sole method to detect 75.2% of them.	mNGS outperformed conventional tests in sensitivity (89.8% vs. 32.5%) and clinical agreement (76.3% vs. 51.3%).	The overall positive detection rate of mNGS regardless of clinical relevance was 69.2% (155/224). mNGS demonstrated higher sensitivity (89.8%) compared to conventional tests (32.5%, *p* < 0.001); higher clinical agreement (76.3% vs. 51.3%, *p* < 0.001).	mNGS: metaDNA-seq (*n* = 223), metaRNA-seq (*n* = 1), and both DNA/RNA sequencing (*n* = 8).	224 blood samples analyzed.	195 pediatric oncology patients with suspected bloodstream infections (BSI).	Retrospective observational study	Wu et al., 2024 [33]
Plasma mNGS influenced clinical management in 13% of cases (14/104), including new or earlier diagnoses in 8 cases. De-escalation of therapy occurred in 28% of cases with positive clinical impact. However, incidental or non-pathogenic organisms were frequently detected, and mNGS led to negative impacts in 4 cases. The study emphasizes the need for cautious interpretation and further prospective validation.	Plasma mNGS showed low concordance with conventional diagnostics. It detected additional organisms not found by standard tests, but many were judged clinically irrelevant or non-causative. Positive percent agreement with conventional diagnostics was 50%; negative agreement was 44%. In some cases, mNGS uniquely identified pathogens missed by other methods, but interpretation was limited by frequent detection of background or non-pathogenic organisms.	Overall agreement between plasma mNGS and conventional diagnostics was 47%. Among confirmed infections, positive percent agreement was 50%; negative agreement in non-infectious cases was 44%. In total, 63.8% of mNGS results identified at least one organism; 33% were concordant with final diagnosis; in 8.5% mNGS uniquely identified the causative pathogen.	Plasma mNGS detected a broad range of organisms, including bacteria, viruses, and fungi., e.g., *P. jirovecii*, *S. aureus*, *E. coli*, *P. aeruginosa*, CMV, EBV, and *Candida spp*. Some detected organisms (e.g., *Torque teno virus*, Anelloviridae) were of unclear clinical significance.	mNGS	104 plasma samples analyzed via mNGS; repeat testing performed in 22 patients (2–4 tests per patient).	71 immunocompromised pediatric patients, including those with hematologic and solid tumors, HCT, and/or solid organ transplantation; tested for indications such as fever, pulmonary syndrome, sepsis, deep-seated, CNS, or musculoskeletal infections.	Retrospective observational study	Lehman et al., 2024 [10]
Limited clinical impact. In 8.5% of cases, mNGS findings alone led to final diagnosis. More commonly provided confirmatory or supplementary data rather than guiding initial diagnosis or therapy. Delayed turnaround (~9 days) reduced utility in acute management.	BAL mNGS showed lower clinical concordance (13.9%) compared to other studies (e.g., 75.6% in adult ICU). Often performed after negative conventional tests, with delayed turnaround. Provided unique diagnostic information in 8.5% of cases. Detected organism types varied by host condition and antimicrobial timing, offering added value in selected subgroups.	BAL mNGS increased diagnostic yield, particularly for viral pathogens. Of 36 tests, 63.8% identified ≥1 organism, but only 13.9% (8/36) were concordant with final ARI diagnosis. Approximately 50% of mNGS results provided additional diagnostic information beyond conventional methods.	Suspected pulmonary infections. mNGS on BAL identified bacterial, viral, and fungal pathogens including *P. aeruginosa*, *Enterobacter cloacae*, *Enterococcus faecium*, CMV, *P. jirovecii*, yeasts, and molds.	mNGS	137 BALF samples collected from 108 patients. Among these, 36 underwent mNGS testing.	108 immunocompromised patients, including hematologic/solid malignancies, aplastic anemia, sickle cell disease with asplenia, and post-HCT.	Single-center retrospective case series	Abraham et al., 2025 [34]
Viral metagenomics improved characterization of viral diversity in FN, identifying clinically relevant viruses, though direct impact on treatment decisions was unclear. Viral composition differed more by sample type than FN diagnosis. Highlights need for further research on clinical utility of viral mNGS in FN management.	Viral metagenomics detected a broader spectrum of viruses than routine PCR. qPCR confirmed key findings (e.g., herpesviruses, polyomaviruses), while nested PCR and Sanger sequencing were used for adenovirus typing. mNGS enabled detection of co-infections and uncommon viruses not routinely tested for.	Viral mNGS demonstrated higher detection rates than standard PCR, including co-infections and rare viruses. Of 1.42 billion post-trimming reads, 21.2% were classified, with 12% of those viral. FN plasma samples had higher viral read counts than swabs.	Viral infections in FN patients: Herpesviridae, Anelloviridae, Adenoviridae, Polyomaviridae; SARS-CoV-2 also detected.	Viral mNGS with Kraken2-based taxonomic classification and qPCR confirmation	Blood and oropharyngeal samples (paired).	15 pediatric patients presenting with febrile neutropenia at admission. Control group: 15 pediatric oncology patients undergoing treatment or in remission.	Case–control study	Sarana da Silva et al., 2025 [35]

ALL—acute lymphoblastic leukemia; AML—acute myeloid leukemia; ARI—acute respiratory infection; BAL/BALF—bronchoalveolar lavage/fluid; BGI—Beijing Genomics Institute (sequencing platform); CMT—conventional microbiological testing; CMV—cytomegalovirus; cfDNA—cell-free DNA; CSF—cerebrospinal fluid; EBV—Epstein–Barr virus; FN—febrile neutropenia; FUO—fever of unknown origin; GM/G-test—galactomannan test/β-D-glucan assay; HAdV—human adenovirus; HCT—hematopoietic cell transplantation; HHV—human herpesvirus; HLH—hemophagocytic lymphohistiocytosis; HRV—human rhinovirus; HSV—herpes simplex virus; ICU—intensive care unit; IL-6—interleukin-6; IFD—invasive fungal disease; LCH—Langerhans cell histiocytosis; mNGS—metagenomic next-generation sequencing; meta-DNA-seq—metagenomic DNA sequencing; meta-RNA-seq—metagenomic RNA sequencing; NHL—non-Hodgkin lymphoma; NGS—next-generation sequencing; NPV—negative predictive value; PPV—positive predictive value; qPCR—quantitative polymerase chain reaction; RB—retinoblastoma; RSV—respiratory syncytial virus; RT-PCR—reverse transcription polymerase chain reaction; RTI—respiratory tract infection; SARS-CoV-2—severe acute respiratory syndrome coronavirus 2; TTV—torque teno virus; UTI — urinary tract infection; VZV—varicella-zoster virus.

**Table 2:** Studies using whole-genome sequencing for infection diagnosis and outbreak investigation in immunocompromised pediatric patients with malignancies.

Clinical Impact	Comparison with Conventional Methods	Diagnostic Performance	Type of Infection/Pathogens Detected	NGS Approach	Sample Type	Patients Population	Study Type	Author
WGS enabled detailed analysis of resistance and virulence genes in *A. baumannii* isolates, supporting infection control efforts and informing future treatment strategies through improved understanding of bacterial transmission.	WGS enabled detailed resistance gene profiling and clonal lineage assignment not achievable with conventional phenotypic or culture-based methods.	Not applicable—study focused on WGS for resistance profiling, not diagnostic detection.	Multidrug-resistant *A. baumannii*.	WGS	Blood (*n* = 16), central venous port blood (*n* = 6), BALF samples (*n* = 6), wound, tissue, and pleural fluid samples (*n* = 1 each).	27 infected pediatric cancer patients with different types of malignancies.	Retrospective genomic surveillance study	Jalal et al., 2021 [36]
WGS findings informed enhanced infection control strategies and supported recommendations for routine genomic surveillance in HCT settings. Enabled detailed outbreak mapping and identification of international HAdV-A31 dissemination.	WGS offered higher resolution than conventional methods, revealing nosocomial transmission and international linkages undetected by standard epidemiology.	WGS enabled high-resolution phylogenetic analysis; 17/20 HAdV-A31 isolates clustered closely (0–8 mutations), indicating outbreak. No formal sensitivity/specificity reported.	Human adenovirus detected in 57 episodes (86% blood PCR positive); WGS identified HAdV-A31 (outbreak strain), HAdV-C1, and HAdV-C2. No evidence of recent transmission based on phylogenetic analysis.	WGS with Illumina MiSeq	Stool culture isolates (*n* = 15), urine culture (*n* = 2), direct WGS from urine (*n* = 2) and stool (*n* = 1).	55 pediatric HCT recipients, with leukemia/lymphoma (43%), solid tumors (24%), immunodeficiency (16%), hematologic disorders (13%), and Hurler syndrome (4%).	Retrospective case series	Fattouh et al., 2022 [37]
WGS confirmed nosocomial SARS-CoV-2 transmission in immunocompromised pediatric patients, supporting recommendations for enhanced infection control and routine genomic surveillance in hospital settings.	WGS provided higher resolution than RT-PCR, enabling identification of transmission links and viral mutations not detectable by standard methods.	WGS revealed high genomic similarity among SARS-CoV-2 isolates, indicating nosocomial transmission. No formal sensitivity or specificity reported.	Viral infection: SARS-CoV-2 (lineage B.1.470).	WGS	Nasopharyngeal swabs.	5 immunocompromised children (AML/Ewing sarcoma, age 1–14), mostly male.	Retrospective case series	Putri et al., 2022 [38]
WGS enabled targeted infection prevention and control investigations and may have prevented further transmission. None of the clusters would have been identified without WGS. This was the first broad, prospective WGS-based bacterial surveillance study in immunocompromised pediatric patients over multiple years.	Conventional surveillance failed to detect any of the 18 WGS-identified transmission clusters. Standard methods relied on clinical suspicion and basic epidemiology, missing silent or indirect transmission events revealed by genomic relatedness.	WGS identified 18 multi-patient transmission clusters among 1497 isolates (1.2% of total), spanning 9 bacterial species. Genomic data enabled detection of transmission with as few as 0–20 allelic differences (cgMLST), enhancing resolution beyond conventional typing.	Healthcare-associated infections, including bloodstream, wound, and catheter-related infections. Detected pathogens included *S. aureus*, *E. coli*, *K. pneumoniae*, *P. aeruginosa*, *E. cloacae*, *Enterococcus faecalis*, and *Pseudomonas putida*.	WGS with core genome multilocus sequence typing (cgMLST)	Clinical diagnostic specimens (inpatient and outpatient), including blood, respiratory tract, urine, skin/soft tissue, and sterile sites; species with ≥3 isolates per year included CoNS excluded.	1025 patients; 1497 bacterial isolates (16 species) obtained from clinical diagnostic specimens as part of a genomic surveillance program.	Prospective observational cohort study	Hakim et al., 2024 [39]

AML—acute myeloid leukemia; BALF—bronchoalveolar lavage fluid; cgMLST—core genome multilocus sequence typing; HAdV—human adenovirus; HCT—hematopoietic cell transplantation; RT-PCR—reverse transcription polymerase chain reaction; SARS-CoV-2—severe acute respiratory syndrome coronavirus 2; WGS—whole-genome sequencing.

**Table 3:** Studies using targeted next-generation sequencing approaches for infection diagnosis in immunocompromised pediatric patients with malignancies.

Clinical Impact	Comparison with Conventional Methods	Diagnostic Performance	Type of Infection/Pathogens Detected	NGS Approach	Sample Type	Patients Population	Study Type	Author
NGS enabled detection of novel and rare viruses (e.g., AstV VA3) not captured by PCR, supporting its potential relevance in HCT patients. Asymptomatic enteric viral presence may predispose to gastrointestinal GVHD and worse outcomes.	ViroCap matched all PCR-confirmed detections and identified viruses missed by PCR, EIA, and ICT, suggesting broader detection range than conventional assays.	ViroCap confirmed viruses detected by clinical PCR (e.g., ADV, NoV) and additionally identified other clinically relevant viruses (e.g., BKV, HRV, HHV-7) missed by routine testing.	Viral infections; detected pathogens included adenoviruses (A, C, AAV), norovirus, BKV, HRV (B, C), KI virus, HHV-7, astrovirus VA3, and alphatorquevirus.	Targeted NGS using ViroCap (hybrid-capture panel covering 34 viral families and 337 species of DNA/RNA viruses).	Stool samples	11 clinical pediatric HCT recipients with gastrointestinal symptoms, suspected of GVHD	Retrospective diagnostic study	Jansen et al., 2020 [40]
Study highlights need to screen HCT patients and donors for HPgV-1 to reduce transmission risk. First-time full-genome characterization of HPgV-1 in this cohort; phylogenetic and intra-host variation analyses provided insight into viral diversity.	TE-mNGS enabled detection and full genome characterization of HPgV-1, which was not achievable by conventional methods.	HPgV-1 detected in 3/14 patients (21.4%) by TE-mNGS; findings confirmed by qRT-PCR. No formal sensitivity or specificity reported.	HPgV-1 detected in 3 patients (day 0 and day 3 samples); CMV detected in 1 patient.	Target enrichment metagenomic NGS (TE-mNGS) using Illumina MiSeq; applied to detect viruses beyond routine qRT-PCR coverage.	Plasma samples from pediatric HCT recipients analyzed to detect human pegivirus-1	14 pediatric patients post-HCT, 13 with febrile neutropenia	Retrospective case series	Ludowyke et al., 2022 [41]

ADV—adenovirus; AstV VA3—astrovirus VA3; BKV—BK virus; EIA—enzyme immunoassay; GVHD—graft-versus-host disease; HCT—hematopoietic cell transplantation; HHV—human herpesvirus; HPgV-1—human pegivirus-1; HRV—human rhinovirus; ICT—immunochromatographic test; NGS—next-generation sequencing; NoV—norovirus; PCT—procalcitonin; TE-mNGS — transcriptome-enriched metagenomic next-generation sequencing.

**Table 4:** Studies using RNA sequencing for host-response profiling in immunocompromised pediatric patients with malignancies.

Clinical Impact	Comparison with Conventional Methods	Diagnostic Performance	Type of Infection/Pathogens Detected	NGS Approach	Sample Type	Patients Population	Study Type	Author
Host transcriptomic profiles distinguished unexplained fever from true infections, suggesting many FUO episodes may not reflect occult infection. Gene expression signatures identifying bacteremia could support targeted antibiotic use in FN patients.	Conventional microbiological tests were often inconclusive; transcriptomic profiling provided discriminatory host-response signatures even in culture-negative episodes.	Transcriptomic analysis distinguished bacteremia and non-bloodstream MDI from unexplained fever via distinct gene expression profiles (e.g., 1206 DEGs in bacteremia vs. unexplained fever); limited discrimination between bacterial and viral MDI.	Bacteremia, non-bloodstream MDI (bacterial/viral), CDI, and unexplained fever. Transcriptomic profiles indicated host responses to pathogens including *S. aureus*, *E. coli*, *Mycobacterium tuberculosis*, *Salmonella*, and *Leishmania*.	RNA-seq	Peripheral blood mononuclear cell (PBMC) samples	64 pediatric patients with solid tumors or leukemia on active treatment; 80 FN episodes included for transcriptomic analysis	Prospective multicenter observational cohort study	Haeusler et at, 2022 [42]
Findings highlight the value of sinus evaluation and molecular diagnostics (e.g., 16S rRNA) in immunocompromised pediatric patients with FUO, supporting targeted therapy. Emphasizes need for expanded diagnostics in resource-limited settings.	Combined phenotypic and molecular methods improved pathogen identification compared to culture alone; enhanced diagnostic precision in sinus infections.	Culture positivity rate was 40% (36/90); molecular methods enabled precise species-level identification of bacterial pathogens.	Paranasal sinus infections (sinusitis); 36 bacterial isolates (40%), including *P. aeruginosa*, *Streptococcus agalactiae*, *S. aureus*, *E. coli*, *K. pneumoniae*, *A. baumannii*, *Nocardia* spp., *S. pneumoniae*, *E. faecium*.	16S rRNA gene sequencing	Paranasal sinus samples (*n* = 90), analyzed using phenotypic and molecular methods.	90 febrile pediatric patients with malignancy and FUO; underlying diseases: ALL (52.2%), Burkitt’s lymphoma (18.9%), aplastic anemia (14.5%), osteosarcoma (7.8%), medulloblastoma (6.7%)	Retrospective observational study	Ghaffari et al., 2024 [43]

ALL—acute lymphoblastic leukemia; CDI—*Clostridioides difficile* infection; FN—febrile neutropenia; FUO—fever of unknown origin; MDI—microbiologically documented infection; PBMC—peripheral blood mononuclear cells; RNA-seq—RNA sequencing.

## Data Availability

All data supporting the findings of this study are available in the cited literature. The extracted dataset used for synthesis can be obtained from the corresponding author upon reasonable request.

## Supplementary Materials

The following supporting information can be downloaded at: https://www.mdpi.com/article/10.3390/jcm14186444/s1, File S1: Systematic review protocol; Table S1: PRISMA 2020 Checklist; Table S2: The complete search strategies for PubMed/MEDLINE, Embase, and Scopus; Table S3: Summary of findings (simplified GRADE assessment of certainty of evidence for key outcomes of NGS in pediatric oncology and HCT patients); Table S4: Comprehensive summary of studies using next-generation sequencing technologies for infection diagnosis in immunocompromised pediatric patients with malignancies; Table S5: Joanna Briggs Institute (JBI) risk-of-bias appraisal for the 24 included studies.

## Abbreviations

The following abbreviations are used in this manuscript:

ADV	adenovirus
AUC	area under the curve
BAL	bronchoalveolar lavage
BALF	bronchoalveolar lavage fluid
BKV	BK virus
BSI	bloodstream infection
CDI	Clostridioides difficile infection
cfDNA	cell-free DNA
CMV	cytomegalovirus
CNS	central nervous system
CVC	central venous catheter
DEGs	differentially expressed genes
EBV	Epstein–Barr virus
EIA	enzyme immunoassay
FIND	Foundation for Innovative New Diagnostics
FN	febrile neutropenia
FUO	fever of unknown origin
GI	gastrointestinal
GM	galactomannan
G-test	β-D-glucan test
GVHD	graft-versus-host disease
HAdV	human adenovirus
HCT	hematopoietic cell transplantation
HHV	human herpesvirus
HPgV-1	human pegivirus-1
HRV	human rhinovirus
ICU	intensive care unit
ICT	immunochromatographic test
IL-6	interleukin-6
JBI	Joanna Briggs Institute
mNGS	metagenomic next-generation sequencing
NGS	next-generation sequencing
NPV	negative predictive value
PCR	polymerase chain reaction
PPV	positive predictive value
PRISMA	Preferred Reporting Items for Systematic Reviews and Meta-Analyses
qPCR	quantitative polymerase chain reaction
qRT-PCR	quantitative reverse transcription PCR
RNA-seq	RNA sequencing
RTI	respiratory tract infection
SARS-CoV-2	severe acute respiratory syndrome coronavirus 2
TPD	traditional pathogen detection
UTI	urinary tract infection
VZV	varicella-zoster virus
WGS	whole-genome sequencing
