# Supplementary material for: Next-Generation Sequencing for Infectious Disease Diagnostics in Pediatric Patients with Malignancies or After Hematopoietic Cell Transplantation: A Systematic Review
Source: J Clin Med. 2025 Sep 12;14(18):6444. doi: 10.3390/jcm14186444 (PMC12470785; doi:10.3390/jcm14186444)
Supplement: Supplementary file 1 [file jcm-14-06444-s001.zip › Supplementary Material File S1.pdf]

Supplementary File S1. Systematic Review Protocol

Title

Next-Generation Sequencing for Infectious Disease Diagnostics in Pediatric Cancer Patients: A Systematic Review

1. Background

Infections remain a major cause of morbidity and mortality among pediatric patients with cancer and those undergoing hematopoietic cell transplantation (HCT). Conventional diagnostic methods, including culture and polymerase chain reaction (PCR), often lack sensitivity, require long turnaround times, and may fail to identify atypical or novel pathogens. Next-generation sequencing (NGS) approaches, including metagenomic NGS (mNGS), targeted sequencing, and ribosomal RNA sequencing (16S/18S/ITS), have emerged as promising tools for comprehensive pathogen detection. This systematic review aims to summarize current evidence regarding the diagnostic yield, clinical utility, and limitations of NGS in this vulnerable population.

2. Objectives and PICO Question

Population (P): Pediatric patients (0–18 years) with cancer, including those undergoing chemotherapy, radiotherapy, or hematopoietic cell transplantation (autologous or allogeneic).

Intervention (I): Use of NGS techniques (e.g., metagenomic NGS, targeted sequencing, 16S/18S/ITS rRNA sequencing, whole-genome sequencing).

Comparison (C): Conventional microbiological diagnostic methods (culture, PCR, antigen testing) or no comparison.

Outcomes (O): Diagnostic yield, turnaround time, impact on patient management, detection of atypical or novel pathogens, and patient-centered outcomes where available.

PICO-based Question:

In children with cancer, how does NGS compare with conventional diagnostics in identifying infectious agents, improving diagnostic yield, and influencing clinical outcomes?

3. Methods

3.1 Design

This systematic review was conducted in accordance with the PRISMA 2020 statement, with relevant elements from the PRISMA-Diagnostic Test Accuracy (DTA) extension incorporated where applicable. A completed PRISMA checklist is provided in Supplementary Table S1, and the PRISMA flow diagram is presented in Figure 1.

3.2 Eligibility Criteria

Inclusion criteria:

- Population: Pediatric patients (aged 0–18 years) with oncological diseases, including those undergoing hematopoietic cell transplantation.
- Intervention: Use of NGS technologies for diagnosis of infections (mNGS, targeted NGS, WGS, 16S/18S/ITS sequencing).
- Outcomes: Diagnostic performance (e.g., pathogen identification, diagnostic yield, turnaround time), clinical relevance (e.g., changes in antimicrobial therapy), and/or patient outcomes.
- Study type: Original research (prospective or retrospective cohorts, case series, clinical trials).
- Language: English.
- Timeframe: January 2010 – April 2025.

Exclusion criteria:

- Studies exclusively involving adult populations or without separate pediatric analyses.
- Studies focusing only on tumor genomics or microbiome composition without infectious disease relevance.
- Reviews, editorials, conference abstracts, commentaries.

3.3 Information Sources and Search Strategy

- Databases: PubMed/MEDLINE, Embase, and Scopus.
- Date range: 1 January 2010 – 22 April 2025.
- Filters: English language, human subjects, pediatric age (0–18 years).
- Publication type: Original research only.
- The complete search strategies are available in Supplementary Table S2.

- No additional searches (reference lists, citation tracking, grey literature) were conducted.

3.4 Study Selection

- Records were imported into Rayyan (web-based tool for systematic review screening).
- Two independent reviewers (A.J., A.S.) screened titles and abstracts, followed by full-text review.
- Disagreements were resolved by consensus or consultation with a third reviewer (J.S.).
- Numbers at each stage are shown in the PRISMA flow diagram (Figure 1).

3.5 Data Extraction

- Two reviewers independently extracted study characteristics (authors, year, country, patient population, sample type, NGS approach, outcomes).
- Extracted outcomes: diagnostic yield, pathogens detected, turnaround time, impact on clinical management, patient outcomes if reported.
- Discrepancies were resolved by discussion.

3.6 Risk of Bias Assessment

- Study quality was evaluated using the Joanna Briggs Institute (JBI) tools for case series and cohort studies.
- QUADAS-2, while the standard for diagnostic accuracy studies, was not consistently applicable due to heterogeneity of designs; this is acknowledged as a limitation.

3.7 Data Synthesis

- Primary synthesis was narrative due to heterogeneity in study designs, outcomes, and reference standards.
- Where feasible, results are summarized as medians, interquartile ranges, and ranges across studies.
- No formal meta-analysis was prespecified.

4. Planned Results Reporting

- General description of included studies.
- Summary of diagnostic yield and comparison with conventional diagnostics.
- Impact of NGS on patient management and outcomes.
- Subgroup analyses by NGS modality and sample type.
- Discussion of strengths, limitations, and implications for research and practice.

5. Bias Minimization Measures

- A priori definition of PICO, eligibility criteria, outcomes, and analysis plan.
- Independent duplicate screening and data extraction.
- Resolution of disagreements by consensus or third-party adjudication.
- Standardized data collection forms.
- Risk of bias assessment using validated tools.

6. Deviations from Protocol

- No major deviations were introduced during the conduct of the review.
- Lack of prospective registration in PROSPERO or similar repository is acknowledged as a limitation.

7. Dissemination

The results of this systematic review are presented in the main manuscript, which was submitted for publication in the *Journal of Clinical Medicine*.
