# Supplementary material for: Next-Generation Sequencing for Infectious Disease Diagnostics in Pediatric Patients with Malignancies or After Hematopoietic Cell Transplantation: A Systematic Review
Source: J Clin Med. 2025 Sep 12;14(18):6444. doi: 10.3390/jcm14186444 (PMC12470785; doi:10.3390/jcm14186444)
Supplement: Supplementary file 1 [file jcm-14-06444-s001.zip › Supplementary Material Table S2.pdf]

Supplementary Table S2. The complete search strategies for PubMed/MEDLINE, Embase, and Scopus

Database	Search Strategy	Date Range	Last Search Date	Filters Applied
**PubMed/ MEDLINE**	("next generation sequencing"[Title/Abstract] OR "NGS"[Title/Abstract] OR "metagenomic sequencing"[Title/Abstract]) AND ("infection"[Title/Abstract] OR "infectious disease"[Title/Abstract] OR "pathogen"[Title/Abstract]) AND ("cancer"[Title/Abstract] OR "oncology"[Title/Abstract] OR "leukemia"[Title/Abstract] OR "lymphoma"[Title/Abstract]) AND ("child"[Title/Abstract] OR "pediatric"[Title/Abstract] OR "paediatric"[Title/Abstract])	Jan 1, 2010 – Apr 22, 2025	Apr 22, 2025	Language: English; Publication type: original research articles (including cohort studies, case series, clinical trials); Humans
**Embase**	('next generation sequencing'/exp OR 'next generation sequencing':ti,ab OR ngs:ti,ab OR 'metagenomic sequencing':ti,ab OR 'metagenomic next-generation sequencing':ti,ab OR 'mngs':ti,ab OR 'whole genome sequencing':ti,ab OR '16s rrna':ti,ab OR '18s rrna':ti,ab OR 'internal transcribed spacer':ti,ab) AND ('infection'/exp OR infection*:ti,ab OR pathogen*:ti,ab OR 'infectious disease*':ti,ab) AND ('cancer patient'/exp OR 'neoplasm'/exp OR cancer*:ti,ab OR oncolog*:ti,ab OR leukemia:ti,ab OR lymphoma:ti,ab OR 'hematopoietic stem cell transplantation':ti,ab) AND ('child'/exp OR pediatric*:ti,ab OR paediatric*:ti,ab OR child*:ti,ab OR infant*:ti,ab OR neonate*:ti,ab OR adolescent*:ti,ab)	Jan 1, 2010 – Apr 22, 2025	Apr 22, 2025	Language: English; Document type: article (excluding conference abstracts); Age: 0–18 years
**Scopus**	(TITLE-ABS-KEY("next generation sequencing" OR ngs OR "metagenomic sequencing" OR mNGS OR "whole genome sequencing" OR "16s rrna" OR "18s rrna" OR "internal transcribed spacer")) AND (TITLE-ABS-KEY(infection OR infections OR pathogen OR "infectious disease")) AND (TITLE-ABS-KEY(cancer OR oncology OR leukemia OR lymphoma OR "hematologic	Jan 1, 2010 – Apr 22, 2025	Apr 22, 2025	Language: English; Document type: article (excluding reviews, conference abstracts, editorials); Subject area: Medicine, Biochemistry, Genetics and

Database	Search Strategy	Date Range	Last Search Date	Filters Applied
	malignancy" OR "hematopoietic stem cell transplantation")) AND (TITLE-ABS-KEY(pediatric OR paediatric OR child OR children OR infant OR neonate OR adolescent))			Molecular Biology, Immunology and Microbiology
