# Supplementary material for: Next-Generation Sequencing for Infectious Disease Diagnostics in Pediatric Patients with Malignancies or After Hematopoietic Cell Transplantation: A Systematic Review
Source: J Clin Med. 2025 Sep 12;14(18):6444. doi: 10.3390/jcm14186444 (PMC12470785; doi:10.3390/jcm14186444)
Supplement: Supplementary file 1 [file jcm-14-06444-s001.zip › Supplementary Material Table S3.pdf]

Supplementary Table S3. Summary of Findings (simplified GRADE assessment of certainty of evidence for key outcomes of NGS in pediatric oncology and HCT patients).

Outcome	Certainty of Evidence	Reasons for Downgrading
**Diagnostic yield of NGS compared with conventional methods**	Low	Observational designs; heterogeneity of patient populations, sample types, and sequencing platforms; lack of consistent reference standards
**Impact of NGS on antimicrobial management**	Very low	Small sample sizes; inconsistent reporting of management changes; risk of bias due to lack of blinding and incomplete outcome data
**Turnaround time of NGS**	Low	Inconsistent definitions of turnaround time across studies; selective reporting; absence of standardized benchmarks
**Patient-centered outcomes (mortality, length of stay, ICU admission)**	Very low	Rarely reported across studies; indirect evidence; reliance on observational designs with substantial heterogeneity
