# Supplementary material for: Next-Generation Sequencing for Infectious Disease Diagnostics in Pediatric Patients with Malignancies or After Hematopoietic Cell Transplantation: A Systematic Review
Source: J Clin Med. 2025 Sep 12;14(18):6444. doi: 10.3390/jcm14186444 (PMC12470785; doi:10.3390/jcm14186444)
Supplement: Supplementary file 1 [file jcm-14-06444-s001.zip › Supplementary Material Table S4.pdf]

Supplementary Table S4. Comprehensive Summary of Studies Using Next-Generation Sequencing Technologies for Infection Diagnosis in Immunocompromised Pediatric Patients with Malignancies.

Author	Study Type	Patients Population	Sample Type	NGS Approach	Type of infection / Pathogens Detected	Diagnostic Performance	Comparison with Conventional Methods	Clinical Impact
Armstrong et al., 2019 [1]	Prospective observational cohort study	40 pediatric hematology, oncology, and stem cell transplant patients at risk for invasive fungal disease (IFD)	Blood samples	cfDNA NGS	Polymicrobial infections detected, including fungal (e.g. *Aspergillus fumigatus*, *Candida spp.*, *Rhizopus delemar*, *Pneumocystis jirovecii*), viral (e.g. CMV, BK virus, HHV-6A, VZV), and bacterial pathogens (e.g. *E. coli*, *Pseudomonas spp.*, *Enterococcus spp.*, *Streptococcus mitis*, *Helicobacter pylori*)	NGS identified fungal pathogens in 7 of 40 high-risk patients and matched conventional diagnostics in 4 of 6 proven IFD cases. Missed detections attributed to limited cfDNA release or uncertain clinical relevance.	NGS showed good concordance with invasive fungal diagnostics (lung, pancreatic, scalp), detecting pathogens at species level. Missed some tissue-only findings (e.g. *R. oryzae*), but identified *P. jirovecii* not found by conventional methods.	cfDNA NGS identified fungal pathogens in some high-risk patients but was not available in real-time and did not influence treatment decisions. Despite negative NGS results, 72% of patients without proven IFD received ≥1 week of antifungal therapy.
Jansen et al., 2020 [2]	Retrospective diagnostic study	11 clinical pediatric HCT recipients with gastrointestinal symptoms, suspected of GVHD	Stool samples	Targeted NGS using ViroCap (hybrid-capture panel covering 34 viral families and 337 species of DNA/RNA viruses	Viral infections; detected pathogens included adenoviruses (A, C, AAV), norovirus, BKV, HRV (B, C), KI virus, HHV-7, astrovirus VA3, and alphatorquevirus	ViroCap confirmed viruses detected by clinical PCR (e.g. ADV, NoV) and additionally identified other clinically relevant viruses (e.g. BKV, HRV, HHV-7) missed by routine testing.	ViroCap matched all PCR-confirmed detections and identified viruses missed by PCR, EIA, and ICT, suggesting broader detection range than conventional assays.	NGS enabled detection of novel and rare viruses (e.g. AstV VA3) not captured by PCR, supporting its potential relevance in HCT patients. Asymptomatic enteric viral presence may predispose to gastrointestinal GVHD and worse outcomes.
Shen et al., 2021 [3]	Prospective observational diagnostic study	70 febrile pediatric patients with hematological disorders; mostly immunocompromised due to chemotherapy, HCT, or underlying disease	Plasma (n=62), throat swabs (n=34), bone marrow (n=4), bronchoalveolar fluid (BALF) (n=4); total 104 samples	mNGS	Bacterial, viral, and fungal pathogens detected by mNGS, including *P. aeruginosa*, *K. pneumoniae*, *S. aureus*, *C. albicans*, EBV, CMV, HSV-1, HHV-7, parvovirus B19, adenovirus, rhinovirus, and polyomaviruses.	mNGS showed a high detection rate (84.6%; 88/104 samples), identifying pathogens in both plasma and respiratory samples, including polymicrobial and rare infections. Enabled pathogen detection even when routine tests were negative.	mNGS detected all pathogens found by conventional tests (5/70; 7.1%) and identified additional clinically relevant microbes in cases with negative routine diagnostics. Enabled overlapping detection across sample types (e.g. blood + swab in 14/34 cases).	mNGS guided antimicrobial adjustment in 55/70 (78.6%) patients, leading to clinical improvement. In 21.4%, results were irrelevant or missed likely pathogens (e.g. *P. jirovecii*, *A. fumigatus*), highlighting both utility and limitations in real-world use.
Horiba et al., 2021 [4]	Retrospective diagnostic cohort study	Plasma/serum samples of 112 pediatric	Plasma/serum samples	mNGS	Putative bacterial pathogens detected in 15/87 culture-negative FN cases; confirmed all	NGS detected pathogens in 5/10 FN patients with positive cultures, 15/87 (17%)	NGS confirmed all culture-positive FN cases and identified additional pathogens in culture-	NGS findings suggest that translocation of oral, skin, and gut microbiota may contribute to FN pathogenesis

		patients with FN and 10 patients with neutropenia without fever			5 culture-positive FN cases. DNA viruses (e.g. CMV, HHV-6B, EBV, TTV) in 19 pts; *Malassezia restricta* in 1 case. Findings suggest flora translocation.	culture-negative FN cases, and 3/8 NE patients. Showed added value in cases with negative standard diagnostics.	negative cases, demonstrating complementary value to conventional diagnostics.	in neutropenic patients. Potential to reveal hidden sources of infection when cultures are negative.
Jalal et al., 2021 [5]	Retrospective genomic surveillance study	27 infected pediatric cancer patients with different types of malignancies	Blood (n=16), central venous port blood (n=6), BALF samples (n=6), wound, tissue, and pleural fluid samples (n=1 each)	WGS	multidrug resistant *A. baumannii*	Not applicable – study focused on WGS for resistance profiling, not diagnostic detection.	WGS enabled detailed resistance gene profiling and clonal lineage assignment not achievable with conventional phenotypic or culture-based methods.	WGS enabled detailed analysis of resistance and virulence genes in *A. baumannii* isolates, supporting infection control efforts and informing future treatment strategies through improved understanding of bacterial transmission.
Zhang et al., 2022 [6]	Retrospective observational diagnostic study	147 pediatric patients with hematologic al malignancies undergoing fever of unknown origin (FUO) (after chemotherap y or HCT)	Blood samples	mNGS	Bacterial, viral, and fungal pathogens detected: most common were *P. aeruginosa*, *K. pneumoniae*, *A. baumannii*, CMV, HHV-1, parvovirus B19, *A. fumigatus*, and *C. parapsilosis*.	mNGS identified pathogens in 76.2% (112/147) of FUO cases with prior negative conventional tests. Pathogens were considered causative in 44.6%, and clinical resolution followed therapy adjustment in 27.9%.	Outperformed conventional methods in FUO by detecting pathogens in cases missed by standard tests; added microbiological value in 27.9% of patients.	mNGS revealed a high rate of coinfections (35.7%), emphasizing the need for careful clinical correlation. Frequent detection of viruses not matching symptoms highlighted the importance of integrating mNGS with host-response markers and clinical judgment.
Haeusler et at, 2022 [7]	Prospective multicenter observational cohort study	64 pediatric patients with solid tumors or leukemia on active treatment; 80 FN episodes included for transcriptom ic analysis	Peripheral blood mononucle ar cell (PBMC) samples	RNA-seq	Bacteraemia, non-bloodstream MDI (bacterial/viral), CDI, and unexplained fever. Transcriptomic profiles indicated host responses to pathogens incl. *S. aureus*, *E. coli*, *Mycobacterium tuberculosis*, *Salmonella*, *Leishmania*.	Transcriptomic analysis distinguished bacteraemia and non-bloodstream MDI from unexplained fever via distinct gene expression profiles (e.g. 1206 DEGs in bacteraemia vs unexplained fever); limited discrimination between bacterial and viral MDI.	Conventional microbiological tests were often inconclusive; transcriptomic profiling provided discriminatory host-response signatures even in culture-negative episodes.	Host transcriptomic profiles distinguished unexplained fever from true infections, suggesting many FUO episodes may not reflect occult infection. Gene expression signatures identifying bacteraemia could support targeted antibiotic use in FN patients.
Qu et al., 2022 [8]	Retrospective observational diagnostic study	101 pediatric recipients after allo-HCT	BALF (n=54), blood (n=32), and cerebrospin	mNGS	mNGS identified pathogens in 91.7% BAL, 85.7% blood, and 73.3% CSF samples; common pathogens:	Higher sensitivity of mNGS vs conventional tests (89.7% vs 21.8%), especially for fungal and viral infections;	Compared to culture and RT-PCR, mNGS detected significantly more pathogens, especially fungi and viruses. mNGS	mNGS supported antibiotic guidance through faster and broader pathogen detection, despite false negatives/positives.

			al fluid (CSF) (n=15) samples		CMV, *P. jiroveci*, *P. aeruginosa*, *K. pneumoniae*, HHV-6B, *Aspergillus*, *Mucor*; mixed infections in 6 BAL and 5 blood cases.	slightly lower specificity (78.5% vs 92.9%).	outperformed conventional methods in BALF, blood, and CSF samples in terms of sensitivity, and enabled detection of mixed and atypical infections.	Interpretation was challenged by sample type (e.g. BALF contamination), delayed processing, and absence of RNA sequencing, limiting viral detection.
Wang et al., 2022 [9]	Retrospective cohort study	55 children with hematologic malignancies and suspected pulmonary infections undergoing bronchoscop y	BALF samples	mNGS	Pulmonary infections with bacterial (e.g. *S. pneumoniae*, *H. influenzae*, *S. aureus*), viral (e.g. CMV, RSV, HPIV3, EBV), and fungal pathogens (e.g. *P. jirovecii*, *A. fumigatus*, *R. oryzae*) detected by mNGS in BALF.	mNGS alone had a higher positivity rate (87.3%) than conventional methods (34.5%, $P < 0.001$); detected bacteria in 31%, viruses in 45.5%, and fungi in 34.5%. Mixed infections identified in 31% of cases. Combined with conventional tests, etiology was established in 91%.	Conventional tests included culture, serology (e.g. RSV, CMV, EBV), and fungal antigen assays (GM/G-test). mNGS detected mixed infections in 30.9% vs 7.3% by conventional methods, especially bacterial-viral and fungal-viral co-infections.	mNGS supported timely and targeted antibiotic adjustments in cases not covered by empirical therapy, potentially improving outcomes. It also enabled de-escalation in selected cases. Authors recommend early BAL mNGS in children with hematologic malignancy and pulmonary infection.
Wang et al., 2022 [10]	Retrospective observational study	258 febrile pediatric patients with leukemia, lymphoma, other malignancies , or HCT; infections included RTI/pneumo nia, FUO, BSI, abdominal, CNS, GI, oral, and soft tissue infections.	Blood (n=157), nasopharyn geal swabs (n=53), BALF (n=30), sputum (n=6), pus (n=5), hydrothora x/ascites (n=4), CSF (n=3)	mNGS	mNGS detected bacteria (e.g. *H. parainfluenzae*, *P. aeruginosa*, *K. pneumoniae*), viruses (e.g. CMV, EBV, HHV-7, HSV-1, parvovirus B19), and fungi (e.g. *P. carinii*, *A. fumigatus*, *C. parapsilosis*, *Fusarium* spp.). Co-infections (bacteria/viruses/fungi) were frequent; >50% of positives involved ≥2 pathogens.	mNGS was positive in 86.8% of cases vs 30.2% with conventional methods; 59.7% of infections were detected by mNGS only. In 71.4% of mNGS-positive cases, pathogens were considered clinically relevant. IL-6 ≥390 pg/mL improved diagnostic precision for bacterial infections in mixed results.	mNGS detected pathogens in 59.7% of cases missed by conventional methods, while only 3.1% were positive by conventional tests alone. Agreement was limited (27.1%), with differing results in 37% of concordant positives. mNGS had higher positivity rates across all sample types, especially NPS (94.3% vs 18.9%) and BALF (96.7% vs 36.7%).	mNGS improved etiologic diagnosis in FUO cases and enabled more targeted antimicrobial decisions. Integration with IL-6 enhanced clinical interpretation of ambiguous or mixed results, supporting precision treatment in immunocompromised children.
Fu et al. 2022 [11]	Retrospective observational stud	70 febrile pediatric patients (median age 5 y) with malignancies or hematologic disorders, including ALL, AML, NHL, LCH, aplastic	Blood, CSF, BALF, sputum, urine, and tissue samples; total n=127 (including 107 blood, 6 CSF, 2 BALF, 3 sputum, 7	DNA-based mNGS (Illumina platform)	Detected bloodstream infections (76%), pneumonia (44.8%), and UTI (2.1%). Most common pathogens: *P. aeruginosa* (20.5%), *K. pneumoniae* (8.7%), CMV (21.3%), and *Candida* spp. (12.6%).	mNGS showed higher sensitivity (91.8%) and NPV (56.3%) than conventional methods (17.7% and 11.4%, respectively), with similar specificity (81.8%). PPV was 97.5% for mNGS vs 88.2% for conventional testing.	mNGS yielded significantly higher positivity than conventional methods (83.3% vs 17.7%, $P < 0.05$); detected pathogens in 80 vs 17 events, respectively.	Early use of mNGS (<48h) in febrile, immunocompromised children was associated with shorter fever duration, lower anti-infective and hospitalization costs. High sensitivity in myelosuppressed patients enabled faster etiologic diagnosis and better-targeted treatment.

		anemia, RB, and Evans syndrome; all underwent mNGS testing.	urine, 2 tissue)					
Fattouh et al. 2022 [12]	Retrospective case series	55 pediatric HCT recipients, with leukemia/lymphoma (43%), solid tumors (24%), immunodeficiency (16%), hematologic disorders (13%), and Hurler syndrome (4%).	Stool culture isolates (n=15), urine culture (n=2), direct WGS from urine (n=2) and stool (n=1)	WGS with Illumina MiSeq	Human adenovirus detected in 57 episodes (86% blood PCR positive); WGS identified HAdV-A31 (outbreak strain), HAdV-C1, and HAdV-C2. No evidence of recent transmission based on phylogenetic analysis.	WGS enabled high-resolution phylogenetic analysis; 17/20 HAdV-A31 isolates clustered closely (0-8 mutations), indicating outbreak. No formal sensitivity/specificity reported.	WGS offered higher resolution than conventional methods, revealing nosocomial transmission and international linkages undetected by standard epidemiology.	WGS findings informed enhanced infection control strategies and supported recommendations for routine genomic surveillance in HCT settings. Enabled detailed outbreak mapping and identification of international HAdV-A31 dissemination.
Ludowyke et al., 2022 [13]	Retrospective case series	14 pediatric patients post-HCT, 13 with febrile neutropenia	Plasma samples from pediatric HCT recipients analyzed to detect Human pegivirus-1	Target enrichment metagenomic NGS (TE-mNGS) using Illumina MiSeq; applied to detect viruses beyond routine qRT-PCR coverage.	HPgV-1 detected in 3 patients (day 0 and day 3 samples); cytomegalovirus (CMV) detected in 1 patient.	HPgV-1 detected in 3/14 patients (21.4%) by target enrichment mNGS; findings confirmed by qRT-PCR. No formal sensitivity or specificity reported.	Target enrichment mNGS enabled detection and full genome characterization of HPgV-1, which was not achievable by conventional methods.	Study highlights need to screen HCT patients and donors for HPgV-1 to reduce transmission risk. First-time full-genome characterization of HPgV-1 in this cohort; phylogenetic and intra-host variation analyses provided insight into viral diversity.
Guo et al, 2022 [14]	Retrospective cohort study	49 children with febrile neutropenia after chemotherapy; majority with leukemia (ALL: 65.3%, AML: 30.6%); 2 cases with HLH.	Plasma (n=49) and BALF (n=12)	mNGS	mNGS detected 70 pathogenic strains in 42 FN cases, including 25 mixed infections. Predominant pathogens included *Aspergillus* spp. and Gram-negative bacteria. *Aspergillus was detected* in 19 cases (often G-test/GM-test negative), and 13/20 Gram-negative bacteria	mNGS showed higher sensitivity (90.9%) but lower specificity (12%) vs TPD (sensitivity 24.7%, specificity 100%). mNGS positivity rate was 85.7% vs 38.8% for TPD (P=0.000); AUC for mNGS was 48.5%.	Outperformed conventional methods in detection rate (63.2% vs 42.5%, P < 0.001); better at identifying mixed and rare infections.	mNGS results influenced clinical management in ~35% of cases, leading to initiation of targeted therapy in 39 patients and de-escalation in 3. Findings support mNGS as a complementary tool, particularly in culture-negative FN, though interpretation requires caution.

					were culture-negative but mNGS-positive.			
Putri et al., 2022 [15]	Retrospective case series	5 immunocompromised children (AML/Ewing sarcoma, age 1-14), mostly male.	Nasopharyngeal swabs	WGS	Viral infection: SARS-CoV-2 (lineage B.1.470)	WGS revealed high genomic similarity among SARS-CoV-2 isolates, indicating nosocomial transmission. No formal sensitivity or specificity reported.	WGS provided higher resolution than RT-PCR, enabling identification of transmission links and viral mutations not detectable by standard methods.	WGS confirmed nosocomial SARS-CoV-2 transmission in immunocompromised pediatric patients, supporting recommendations for enhanced infection control and routine genomic surveillance in hospital settings.
Wu et al., 2023 [16]	Retrospective multicenter study	7 patients with adenovirus infection after haploidentical HCT (from cohort of 976); 6 with acute leukemia, 1 with aplastic anemia; 5 male, 2 female.	Blood, CSF, urine, and BALF samples.	mNGS	Systemic human adenovirus (HAdV) infection involving blood, urine, CSF, and lungs; clinical manifestations included ADV hepatitis and encephalitis.	mNGS successfully identified HAdV infections post-haplo-HCT; no formal sensitivity or specificity metrics reported.	mNGS offers a comprehensive diagnostic approach and may detect infections missed by conventional methods; no direct comparison or statistical analysis reported.	Early HAdV detection by mNGS may enable timely antiviral treatment and improve outcomes. Highlights the value of mNGS in diagnosing adenovirus infections in immunocompromised pediatric patients post-haplo-HCT.
Zhang et al., 2023 [17]	Retrospective observational study	67 pediatric patients with hematological diseases, mostly ALL (52.2%) and AML (14.9%); 28.4% post-HCT; 39 males.	96 specimens: plasma (n=71), CSF (n=11), sputum (n=8), BALF (n=2), hydrothorax, urine, liver biopsy, and abscess fluid (n=1 each).	mNGS	Infections included sepsis, respiratory tract infections, and febrile neutropenia with suspected bloodstream infection. mNGS identified pathogens in 85/96 specimens (88.5%), including simple bacterial (43.5%), fungal (19.4%), viral (11.3%) and mixed infections (25.8%). Frequent pathogens: *K. pneumoniae*, *P. aeruginosa*, *A. baumannii*, *E. coli*, *Y. pneumoniae*, *Aspergillus flavus*, and human herpesviruses.	mNGS showed higher positivity for bacteria and fungi (57.2%) compared to culture (12.5%, $P < 0.01$); detected bacteria (n=27), fungi (n=12), viruses (n=7), and mixed infections (n=16). No fungi were detected by culture.	mNGS showed higher and earlier pathogen detection than culture, identifying organisms missed by conventional methods. In one case, *K. pneumoniae* was confirmed by culture three days after mNGS detection.	mNGS guided treatment adjustments (e.g. linezolid initiation, antifungal addition, antibiotic de-escalation), with most patients improving clinically. Enabled early differentiation between infectious and non-infectious fevers.
Ghaffari et al., 2024 [18]	Retrospective observational study	90 febrile pediatric patients with malignancy and FUO;	Paranasal sinus samples (n=90), analyzed	16S rRNA gene sequencing	Paranasal sinus infections (sinusitis); 36 bacterial isolates (40%), including *P. aeruginosa*, *S. agalactiae*, *S. aureus*,	Culture positivity rate was 40% (36/90); molecular methods enabled precise species-	Combined phenotypic and molecular methods improved pathogen identification compared to culture alone;	Findings highlight the value of sinus evaluation and molecular diagnostics (e.g. 16S rRNA) in immunocompromised

		underlying diseases: ALL (52.2%), Burkitt's lymphoma (18.9%), aplastic anemia (14.5%), osteosarcoma (7.8%), medulloblastoma (6.7%)	using phenotypic and molecular methods.		*E. coli*, *K. pneumoniae*, *A. baumannii*, *Nocardia* spp., *S. pneumoniae*, *E. faecium*.	level identification of bacterial pathogens.	enhanced diagnostic precision in sinus infections	pediatric patients with FUO, supporting targeted therapy. Emphasizes need for expanded diagnostics in resource-limited settings.
Hakim et al, 2024 [19]	Prospective observational cohort study	1025 patients; 1497 bacterial isolates (16 species) obtained from clinical diagnostic specimens as part of a genomic surveillance program.	Clinical diagnostic specimens (inpatient and outpatient), including blood, respiratory tract, urine, skin/soft tissue, and sterile sites; species with ≥3 isolates per year included CoNS excluded.	WGS with core genome multilocus sequence typing (cgMLST)	Healthcare-associated infections, including bloodstream, wound, and catheter-related infections. Detected pathogens included *S. aureus*, *E. coli*, *K. pneumoniae*, *P. aeruginosa*, *E. cloacae*, *E. faecalis*, and *P. putida*.	WGS identified 18 multi-patient transmission clusters among 1497 isolates (1.2% of total), spanning 9 bacterial species. Genomic data enabled detection of transmission with as few as 0-20 allelic differences (cgMLST), enhancing resolution beyond conventional typing.	Conventional surveillance failed to detect any of the 18 WGS-identified transmission clusters. Standard methods relied on clinical suspicion and basic epidemiology, missing silent or indirect transmission events revealed by genomic relatedness.	WGS enabled targeted infection prevention and control investigations and may have prevented further transmission. None of the clusters would have been identified without WGS. This was the first broad, prospective WGS-based bacterial surveillance study in immunocompromised pediatric patients over multiple years.
Xu et al, 2024 [20]	Retrospective cohort study	48 immunocompromised pediatric patients (avg. age 88 mo), mainly with leukemia (52.1%) and solid tumors (20.8%). Most common symptoms: fever (84.9%), cough (29.4%), convulsions	119 clinical samples, including BALF, CSF, blood, stool, peritoneal fluid, pleural fluid, pus, sputum, and swabs; 48 samples from immunocompromised patients.	DNA mNGS (BGI platform)	Detected Gram-positive and Gram-negative bacteria (e.g. *Streptococcus pneumoniae*, *Klebsiella pneumoniae*), Herpesviridae viruses (*CMV*, *EBV*, *HSV*), respiratory viruses (*RSV*, *HPIV*, *HRV*), and fungi including *Pneumocystis jirovecii* and *Candida albicans*. Co-infections were common in immunocompromised children.	mNGS positivity rate: 76.5%, significantly higher than conventional testing (55.5%, P = 0.0006). Positive percent agreement was higher in immunocompromised patients (95.2%) vs immunocompetent (77.8%).	mNGS showed superior pathogen detection compared to conventional microbiological testing (CMT), with a significantly higher positivity rate (76.5% vs 55.5%,. mNGS identified additional pathogens missed by CMT, especially in mixed infections and among immunocompromised patients. In some cases, mNGS provided the only microbiologic evidence of infection.	mNGS influenced clinical diagnosis in 91.7% and led to treatment modifications in 95.8% of immunocompromised patients. The study highlights the value of mNGS for rapid, comprehensive pathogen detection in critically ill immunocompromised children, where infections may be atypical or otherwise undiagnosed.

		(2.1%). Total of 119 samples analyzed.						
Wu et al., 2024 [21]	Retrospective observational study	195 pediatric oncology patients with suspected bloodstream infections (BSI)	224 blood samples analyzed	mNGS:: metaDNA-seq (n=223), metaRNA-seq (n=1), and both DNA/RNA sequencing (n=8).	The overall positive detection rate of mNGS regardless of clinical relevance was 69.2% (155/224). mNGS demonstrated higher sensitivity (89.8%) compared to conventional tests (32.5%, $P < 0.001$); higher clinical agreement (76.3% vs. 51.3%, $P < 0.001$)	mNGS outperformed conventional tests in sensitivity (89.8% vs. 32.5%) and clinical agreement (76.3% vs. 51.3%).	mNGS significantly outperformed conventional microbiological tests (culture, PCR for EBV/CMV, GM/G tests) in sensitivity, clinical agreement, and pathogen coverage—especially for viruses. While both methods were positive in 25.4% of samples, only 15.8% showed identical pathogen identification. mNGS detected 94.5% of clinician-confirmed pathogens, including 100% of viruses, and was the sole method to detect 75.2% of them.	mNGS guided treatment changes (54.3%), improved diagnostic confidence, reduced unnecessary antibiotics, and enabled precise therapy in pediatric cancer patients with suspected BSI.
Lehman et al., 2024 [22]	Retrospective observational study	71 immunocompromised pediatric patients, including those with hematologic and solid tumors, HCT, and/or solid organ transplantation; tested for indications such as fever, pulmonary syndrome, sepsis, deep-seated, CNS, or musculoskeletal infections.	104 plasma samples analyzed via mNGS; repeat testing performed in 22 patients (2-4 tests per patient).	mNGS	Plasma mNGS detected a broad range of organisms, including bacteria, viruses, and fungi., e.g. *Pneumocystis jirovecii*, *Staphylococcus aureus*, *Escherichia coli*, *Pseudomonas aeruginosa*, *CMV*, *EBV*, and *Candida* spp. Some detected organisms (e.g. *Torque teno virus*, *Anelloviridae*) were of unclear clinical significance.	Overall agreement between plasma mNGS and conventional diagnostics was 47%. Among confirmed infections, positive percent agreement was 50%; negative agreement in non-infectious cases was 44%. In total, 63.8% of mNGS results identified at least one organism; 33% were concordant with final diagnosis; in 8.5% mNGS uniquely identified the causative pathogen.	Plasma mNGS showed low concordance with conventional diagnostics. It detected additional organisms not found by standard tests, but many were judged clinically irrelevant or non-causative. Positive percent agreement with conventional diagnostics was 50%; negative agreement was 44%. In some cases, mNGS uniquely identified pathogens missed by other methods, but interpretation was limited by frequent detection of background or non-pathogenic organisms.	Plasma mNGS influenced clinical management in 13% of cases (14/104), including new or earlier diagnoses in 8 cases. De-escalation of therapy occurred in 28% of cases with positive clinical impact. However, incidental or non-pathogenic organisms were frequently detected, and mNGS led to negative impacts in 4 cases. The study emphasizes the need for cautious interpretation and further prospective validation.
Abraham et al., 2025 [23]	Single-center retrospective case series	108 immunocompromised	137 BALF samples collected	mNGS	Suspected pulmonary infections. mNGS on BAL identified	BAL mNGS increased diagnostic yield, particularly for viral	BAL mNGS showed lower clinical concordance (13.9%)	Limited clinical impact. In 8.5% of cases, mNGS findings alone led to final diagnosis.

		patients, including hematologic/ solid malignancies, aplastic anemia, sickle cell disease with asplenia, and post-HCT.	from 108 patients. Among these, 36 underwent mNGS testing.		bacterial, viral, and fungal pathogens including *Pseudomonas aeruginosa*, *Enterobacter cloacae*, *Enterococcus faecium*, *CMV*, *Pneumocystis jirovecii*, yeasts, and molds.	pathogens. Of 36 tests, 63.8% identified ≥1 organism, but only 13.9% (8/36) were concordant with final ARI diagnosis. Approximately 50% of mNGS results provided additional diagnostic information beyond conventional methods.	compared to other studies (e.g., 75.6% in adult ICU). Often performed after negative conventional tests, with delayed turnaround. Provided unique diagnostic information in 8.5% of cases. Detected organism types varied by host condition and antimicrobial timing, offering added value in selected subgroups.	More commonly provided confirmatory or supplementary data rather than guiding initial diagnosis or therapy. Delayed turnaround (~9 days) reduced utility in acute management.
Sarana da Silva et al., 2025 [24]	Case-control study	15 pediatric patients presenting with febrile neutropenia at admission. Control group: 15 pediatric oncology patients undergoing treatment or in remission.	Blood and oropharyngeal samples (paired).	Viral mNGS with Kraken2-based taxonomic classification and qPCR confirmation	Viral infections in FN patients: *Herpesviridae*, *Anelloviridae*, *Adenoviridae*, *Polyomaviridae*; SARS-CoV-2 also detected.	Viral mNGS demonstrated higher detection rates than standard PCR, including co-infections and rare viruses. Of 1.42 billion post-trimming reads, 21.2% were classified, with 12% of those viral. FN plasma samples had higher viral read counts than swabs.	Viral metagenomics detected a broader spectrum of viruses than routine PCR. qPCR confirmed key findings (e.g., herpesviruses, polyomaviruses), while nested PCR and Sanger sequencing were used for adenovirus typing. mNGS enabled detection of co-infections and uncommon viruses not routinely tested.	Viral metagenomics improved characterization of viral diversity in FN, identifying clinically relevant viruses, though direct impact on treatment decisions was unclear. Viral composition differed more by sample type than FN diagnosis. Highlights need for further research on clinical utility of viral mNGS in FN management.

NGS – next-generation sequencing; mNGS – metagenomic next-generation sequencing; WGS –whole-genome sequencing; RNA-seq – RNA sequencing; tNGS – targeted next-generation sequencing; TE-mNGS- target-enrichment metagenomic NGS; cfDNA- cell-free DNA; BAL / BALF – bronchoalveolar lavage / fluid; CSF – cerebrospinal fluid; PBMC – peripheral blood mononuclear cells; FN – febrile neutropenia; HCT – hematopoietic cell transplantation; ALL/ AML –- acute lymphoblastic leukemia / acute myeloid leukemia; NHL – non-Hodgkin lymphoma; RB – retinoblastoma; LCH – Langerhans cell histiocytosis; GVHD – graft-versus-host disease; ADV / BKV / EBV / CMV / HSV / HHV – adenovirus / BK virus / Epstein-Barr virus / cytomegalovirus / herpes simplex virus / human herpesvirus; HPgV-1 – human pegivirus-1; HAdV – human adenovirus; SARS-CoV-2 – severe acute respiratory syndrome coronavirus 2; IFD – invasive fungal disease; TPD – traditional pathogen detection; qPCR / qRT-PCR – quantitative (reverse transcription) polymerase chain reaction; EIA / ICT – enzyme immunoassay / immunochromatographic test; GM / G-test – galactomannan test / β-D-glucan assay; IL-6 – interleukin-6; NPV / PPV – negative predictive value / positive predictive value; AUC – area under the curve; DEGs – differentially expressed genes; CDI –Clostridioides difficile infection; UTI – urinary tract infection; RTI – respiratory tract infection; FUO – fever of unknown origin; BSI – bloodstream infection; CNS – central nervous system; GI – gastrointestinal; ICU – intensive care unit

References

[1] Armstrong AE, Rossoff J, Hollemon D, Hong DK, Muller WJ, Chaudhury S. Cell-free DNA next-generation sequencing successfully detects infectious pathogens in pediatric oncology and hematopoietic stem cell transplant patients at risk for invasive fungal disease. Pediatr Blood Cancer 2019;66:e27734. https://doi.org/10.1002/pbc.27734.
[2] Jansen SA, Nijhuis W, Leavis HL, Riezebos-Brilman A, Lindemans CA, Schuurman R. Broad Virus Detection and Variant Discovery in Fecal Samples of Hematopoietic Transplant Recipients Using Targeted Sequence Capture Metagenomics. Front Microbiol 2020;11:560179. https://doi.org/10.3389/fmicb.2020.560179.
[3] Shen H, Shen D, Song H, Wu X, Xu C, Su G, et al. Clinical assessment of the utility of metagenomic next-generation sequencing in pediatric patients of hematology department. Int J Lab Hematol 2021;43:244–9. https://doi.org/10.1111/ijlh.13370.
[4] Horiba K, Torii Y, Okumura T, Takeuchi S, Suzuki T, Kawada J, et al. Next-Generation Sequencing to Detect Pathogens in Pediatric Febrile Neutropenia: A Single-Center Retrospective Study of 112 Cases. Open Forum Infect Dis 2021;8:ofab223. https://doi.org/10.1093/ofid/ofab223.
[5] Jalal D, Elzayat MG, Diab AA, El-Shqanqery HE, Samir O, Bakry U, et al. Deciphering Multidrug-Resistant Acinetobacter baumannii from a Pediatric Cancer Hospital in Egypt. mSphere n.d.;6:e00725-21. https://doi.org/10.1128/mSphere.00725-21.
[6] Zhang P, Zhang Z-H, Liang J, Shen D-Y, Li J, Wang D, et al. Metagenomic next-generation sequencing for the diagnosis of fever of unknown origin in pediatric patients with hematological malignancy. Clin Chim Acta Int J Clin Chem 2022;537:133–9. https://doi.org/10.1016/j.cca.2022.10.008.
[7] Haeusler GM, Garnham AL, Li-Wai-Suen CS, Clark JE, Babl FE, Allaway Z, et al. Blood transcriptomics identifies immune signatures indicative of infectious complications in childhood cancer patients with febrile neutropenia. Clin Transl Immunol 2022;11:e1383. https://doi.org/10.1002/cti2.1383.
[8] Qu Y, Ding W, Liu S, Wang X, Wang P, Liu H, et al. Metagenomic Next-Generation Sequencing vs. Traditional Pathogen Detection in the Diagnosis of Infection After Allogeneic Hematopoietic Stem Cell Transplantation in Children. Front Microbiol 2022;13:868160. https://doi.org/10.3389/fmicb.2022.868160.
[9] Wang D, Wang W, Ding Y, Tang M, Zhang L, Chen J, et al. Metagenomic Next-Generation Sequencing Successfully Detects Pulmonary Infectious Pathogens in Children With Hematologic Malignancy. Front Cell Infect Microbiol 2022;12. https://doi.org/10.3389/fcimb.2022.899028.
[10] Wang D, Lai M, Song H, Zhang J-Y, Zhao F-Y, Liang J, et al. Integration of Interleukin-6 Improves the Diagnostic Precision of Metagenomic Next-Generation Sequencing for Infection in Immunocompromised Children. Front Microbiol 2022;13:819467. https://doi.org/10.3389/fmicb.2022.819467.
[11] .... Fu Y, Zhu X, Cao P, Shen C, Qian X, Miao H, et al. Metagenomic Next-Generation Sequencing in the Diagnosis of Infectious Fever During Myelosuppression Among Pediatric Patients with Hematological and Neoplastic Diseases. Infect Drug Resist 2022;15:5425–34. https://doi.org/10.2147/IDR.S379582.
[12] Fattouh R, Stapleton PJ, Eshaghi A, Thomas AD, Science ME, Schechter T, et al. A Prolonged Outbreak of Human Adenovirus A31 (HAdV-A31) Infection on a Pediatric Hematopoietic Stem Cell Transplantation

Ward with Whole Genome Sequencing Evidence of International Linkages. J Clin Microbiol 2022;60:e00665-22. https://doi.org/10.1128/jcm.00665-22.
[13] Ludowyke N, Phumiphanjarphak W, Apiwattanakul N, Manopwisedjaroen S, Pakakasama S, Sensorn I, et al. Target Enrichment Metagenomics Reveals Human Pegivirus-1 in Pediatric Hematopoietic Stem Cell Transplantation Recipients. Viruses 2022;14:796. https://doi.org/10.3390/v14040796.
[14] Guo F, Kang L, Zhang L. mNGS for identifying pathogens in febrile neutropenic children with hematological diseases. Int J Infect Dis IJID Off Publ Int Soc Infect Dis 2022;116:85–90. https://doi.org/10.1016/j.ijid.2021.12.335.
[15] Putri ND, Johar E, Dewi YP, Indrasari ND, Wulandari D, Br Pasaribu MM, et al. Whole-Genome Sequencing of SARS-CoV-2 Infection in a Cluster of Immunocompromised Children in Indonesia. Front Med 2022;9:835998. https://doi.org/10.3389/fmed.2022.835998.
[16] Wu Q, Wu Y, Zhao Y, Zhang Y, Cao J, Wu D, et al. Adenovirus infection diagnosed by metagenomic next-generation sequencing after haploidentical hematopoietic stem cell transplantation: A multicenter study in China. Transpl Infect Dis Off J Transplant Soc 2023;25:e14054. https://doi.org/10.1111/tid.14054.
[17] Y Z, D Z, H X, J W, H Y, L X, et al. Metagenomic next-generation sequencing for detection of pathogens in children with hematological diseases complicated with infection. Mol Cell Probes 2023;67. https://doi.org/10.1016/j.mcp.2022.101889.
[18] ....Ghaffari K, Falahati V, Motallebirad T, Safarabadi M, Tashakor AH, Azadi D. Microbiological and Molecular Study of Paranasal Sinus Infections of Children with Malignancy and Unknown Origin Fever in Markazi Province, Iran. Curr Ther Res 2024;100:100745. https://doi.org/10.1016/j.curtheres.2024.100745.
[19] Hakim H, Glasgow HL, Brazelton JN, Gilliam CH, Richards L, Hayden RT. A prospective bacterial whole-genome-sequencing-based surveillance programme for comprehensive early detection of healthcare-associated infection transmission in paediatric oncology patients. J Hosp Infect 2024;143:53–63. https://doi.org/10.1016/j.jhin.2023.10.015.
[20] Xu, X.; Zheng, Y.; Zhang, X.; Zhang, C.; Gai, W.; Yang, Z. Utility of Metagenomic Next-Generation Sequencing for Diagnosis of Infectious Diseases in Critically Ill Immunocompromised Pediatric Patients. Infect. Drug Resist. 2024, 17, 3579–3591. https://doi.org/10.2147/IDR.S472129.
[21] ... Wu J, Song W, Yan H, Luo C, Hu W, Xie L, et al. Metagenomic next-generation sequencing in detecting pathogens in pediatric oncology patients with suspected bloodstream infections. Pediatr Res 2024;95:843–51. https://doi.org/10.1038/s41390-023-02776-y.
[22] Lehman AC, Goren LR, Evans MD, Toles O, Drozdov D, Andrews SL, et al. Clinical Performance of Plasma Metagenomic Sequencing in Immunocompromised Pediatric Patients. J Pediatr Infect Dis Soc 2024;13:276–81. https://doi.org/10.1093/jpids/piae024.
[23] Abraham A, Green A, Ferrolino J, Flerlage T, Gowen A, Allison KJ, et al. Utility and Safety of Bronchoalveolar Lavage for Diagnosis and Management of Respiratory Illnesses in Immunocompromised Children. J Pediatr Infect Dis Soc 2025;14:piaf015. https://doi.org/10.1093/jpids/piaf015.

[24] Sarana da Silva A, de Campos GM, Altizani GM, de Carvalho E, Barros AC, Cella E, et al. Utilizing Viral Metagenomics to Characterize Pathogenic and Commensal Viruses in Pediatric Patients with Febrile Neutropenia. Viruses 2025;17:345. https://doi.org/10.3390/v17030345.
