# Supplementary material for: Next-Generation Sequencing for Infectious Disease Diagnostics in Pediatric Patients with Malignancies or After Hematopoietic Cell Transplantation: A Systematic Review
Source: J Clin Med. 2025 Sep 12;14(18):6444. doi: 10.3390/jcm14186444 (PMC12470785; doi:10.3390/jcm14186444)
Supplement: Supplementary file 1 [file jcm-14-06444-s001.zip › Supplementary Material Table S5.pdf]

Supplementary Table S5. Joanna Briggs Institute (JBI) Risk-of-Bias Appraisal for the 24 Included Studies

**Author (Year)**	**Study design**	**JBI overall rating**	**Key bias domains flagged**
Armstrong et al., 2019 [1]	Prospective cohort	Moderate	Lack of blinding; small sample; single-center; potential selection, confounding
Jansen et al., 2020 [2]	Retrospective diagnostic	High-Moderate	Convenience sampling; no blinding; single-center; very small sample; incomplete reference standard
Shen et al., 2021 [3]	Prospective cohort	Moderate	Lack of blinding; subjective interpretation; single-center; clinical convenience sampling
Horiba et al., 2021 [4]	Retrospective observational cohort	High-Moderate	Retrospective; no blinding; low sensitivity reference (blood culture); subjective interpretation; single-center
Jalal et al., 2021 [5]	Retrospective genomic cohort	High	Retrospective; no blinding; convenience sampling; small sample; no clinical outcome link
Zhang et al., 2022 [6]	Retrospective observational cohort	High-Moderate	Retrospective; broad fever of unknown origin definition; limited sensitivity reference standard; no blinding, single-center, small sample
Haeusler et al., 2022 [7]	Prospective cohort	Moderate	No blinding; incomplete follow-up; single-center; selection bias
Qu et al., 2022 [8]	Retrospective observational cohort	Moderate	Retrospective; no blinding; low

			sensitivity reference; incomplete data; single-center
Wang et al., 2022 [9]	Retrospective observational cohort	Moderate	Retrospective; no blinding; low sensitivity reference; incomplete data; single-center
Wang et al., 2022 [10]	Retrospective observational cohort	High-Moderate	Retrospective; no blinding; low sensitivity reference; incomplete data; single-center
Fu et al., 2022 [11]	Retrospective observational cohort	High-Moderate	Retrospective; no blinding; low sensitivity reference; incomplete data; single-center
Fattouh et al., 2022 [12]	Case series	High-Moderate	Retrospective; no blinding; convenience sampling; incomplete whole genome sequencing; single-center; descriptive only
Ludowyke et al., 2022 [13]	Case series	High-Moderate	Retrospective; convenience sampling; no blinding; very small sample; no clinical impact; single-center
Guo et al., 2022 [14]	Retrospective observational cohort	High-Moderate	Retrospective; low specificity; no blinding, moderate sample, potential confounding variables
Putri et al., 2022 [15]	Case series	High-Moderate	Convenience sampling; no blinding; small sample; incomplete cluster data; descriptive only
Wu et al., 2023 [16]	Retrospective multicenter	High-Moderate	Small sample; no reference standard, retrospective, convenience sampling
Zhang et al., 2023 [17]	Retrospective observational cohort	High-Moderate	Retrospective; no blinding; low

			sensitivity reference; single center, convenience sampling
Ghaffari et al., 2024 [18]	Retrospective observational cohort	Moderate	Culture vs 16S only; single center; no blinding, convenience sampling, partial reference standard
Hakim et al., 2024 [19]	Prospective surveillance cohort	Low-Moderate	No blinding; no gold-standard reference; single-center
Xu et al., 2024 [20]	Retrospective observational cohort	Moderate	Large sample; no blinding; single center
Wu et al., 2024 [21]	Retrospective observational cohort	Moderate	Large sample; single center; no blinding; low sensitivity reference standard
Lehman et al., 2024 [22]	Retrospective observational cohort	High	Retrospective; no blinding; no reference standard; incomplete data; limited clinical impact
Abraham et al., 2025 [23]	Retrospective case series	Moderate	Retrospective; no blinding; convenience sampling; no reference standard; clinical interpretation bias
Sarana da Silva et al., 2025 [24]	Case-control study	Moderate	Small sample; convenience sampling; no blinding; incomplete data; no clinical outcome

References

[1] Armstrong AE, Rossoff J, Hollemon D, Hong DK, Muller WJ, Chaudhury S. Cell-free DNA next-generation sequencing successfully detects infectious pathogens in pediatric oncology and hematopoietic stem cell transplant patients at risk for invasive fungal disease. Pediatr Blood Cancer 2019;66:e27734. https://doi.org/10.1002/pbc.27734.

[2] Jansen SA, Nijhuis W, Leavis HL, Riezebos-Brilman A, Lindemans CA, Schuurman R. Broad Virus Detection and Variant Discovery in Fecal Samples of Hematopoietic Transplant Recipients

Using Targeted Sequence Capture Metagenomics. Front Microbiol 2020;11:560179. https://doi.org/10.3389/fmicb.2020.560179.
[3] Shen H, Shen D, Song H, Wu X, Xu C, Su G, et al. Clinical assessment of the utility of metagenomic next-generation sequencing in pediatric patients of hematology department. Int J Lab Hematol 2021;43:244–9. https://doi.org/10.1111/ijlh.13370.
[4] Horiba K, Torii Y, Okumura T, Takeuchi S, Suzuki T, Kawada J, et al. Next-Generation Sequencing to Detect Pathogens in Pediatric Febrile Neutropenia: A Single-Center Retrospective Study of 112 Cases. Open Forum Infect Dis 2021;8:ofab223. https://doi.org/10.1093/ofid/ofab223.
[5] Jalal D, Elzayat MG, Diab AA, El-Shqanqery HE, Samir O, Bakry U, et al. Deciphering Multidrug-Resistant Acinetobacter baumannii from a Pediatric Cancer Hospital in Egypt. mSphere n.d.;6:e00725-21. https://doi.org/10.1128/mSphere.00725-21.
[6] Zhang P, Zhang Z-H, Liang J, Shen D-Y, Li J, Wang D, et al. Metagenomic next-generation sequencing for the diagnosis of fever of unknown origin in pediatric patients with hematological malignancy. Clin Chim Acta Int J Clin Chem 2022;537:133–9. https://doi.org/10.1016/j.cca.2022.10.008.
[7] Haeusler GM, Garnham AL, Li-Wai-Suen CS, Clark JE, Babl FE, Allaway Z, et al. Blood transcriptomics identifies immune signatures indicative of infectious complications in childhood cancer patients with febrile neutropenia. Clin Transl Immunol 2022;11:e1383. https://doi.org/10.1002/cti2.1383.
[8] Qu Y, Ding W, Liu S, Wang X, Wang P, Liu H, et al. Metagenomic Next-Generation Sequencing vs. Traditional Pathogen Detection in the Diagnosis of Infection After Allogeneic Hematopoietic Stem Cell Transplantation in Children. Front Microbiol 2022;13:868160. https://doi.org/10.3389/fmicb.2022.868160.
[9] Wang D, Wang W, Ding Y, Tang M, Zhang L, Chen J, et al. Metagenomic Next-Generation Sequencing Successfully Detects Pulmonary Infectious Pathogens in Children With Hematologic Malignancy. Front Cell Infect Microbiol 2022;12. https://doi.org/10.3389/fcimb.2022.899028.
[10] Wang D, Lai M, Song H, Zhang J-Y, Zhao F-Y, Liang J, et al. Integration of Interleukin-6 Improves the Diagnostic Precision of Metagenomic Next-Generation Sequencing for Infection in Immunocompromised Children. Front Microbiol 2022;13:819467. https://doi.org/10.3389/fmicb.2022.819467.
[11]Fu Y, Zhu X, Cao P, Shen C, Qian X, Miao H, et al. Metagenomic Next-Generation Sequencing in the Diagno
[12] Fattouh R, Stapleton PJ, Eshaghi A, Thomas AD, Science ME, Schechter T, et al. A Prolonged Outbreak of Human Adenovirus A31 (HAdV-A31) Infection on a Pediatric Hematopoietic Stem

Cell Transplantation Ward with Whole Genome Sequencing Evidence of International Linkages. J Clin Microbiol 2022;60:e00665-22. https://doi.org/10.1128/jcm.00665-22.
[13] Ludowyke N, Phumiphanjarphak W, Apiwattanakul N, Manopwisedjaroen S, Pakakasama S, Sensorn I, et al. Target Enrichment Metagenomics Reveals Human Pegivirus-1 in Pediatric Hematopoietic Stem Cell Transplantation Recipients. Viruses 2022;14:796. https://doi.org/10.3390/v14040796.
[14] Guo F, Kang L, Zhang L. mNGS for identifying pathogens in febrile neutropenic children with hematological diseases. Int J Infect Dis IJID Off Publ Int Soc Infect Dis 2022;116:85–90. https://doi.org/10.1016/j.ijid.2021.12.335.
[15] Putri ND, Johar E, Dewi YP, Indrasari ND, Wulandari D, Br Pasaribu MM, et al. Whole-Genome Sequencing of SARS-CoV-2 Infection in a Cluster of Immunocompromised Children in Indonesia. Front Med 2022;9:835998. https://doi.org/10.3389/fmed.2022.835998.
[16] Wu Q, Wu Y, Zhao Y, Zhang Y, Cao J, Wu D, et al. Adenovirus infection diagnosed by metagenomic next-generation sequencing after haploidentical hematopoietic stem cell transplantation: A multicenter study in China. Transpl Infect Dis Off J Transplant Soc 2023;25:e14054. https://doi.org/10.1111/tid.14054.
[17] Y Z, D Z, H X, J W, H Y, L X, et al. Metagenomic next-generation sequencing for detection of pathogens in children with hematological diseases complicated with infection. Mol Cell Probes 2023;67. https://doi.org/10.1016/j.mcp.2022.101889.
[18]Ghaffari K, Falahati V, Motallebirad T, Safarabadi M, Tashakor AH, Azadi D. Microbiological and Molecula
[19] Hakim H, Glasgow HL, Brazelton JN, Gilliam CH, Richards L, Hayden RT. A prospective bacterial whole-genome-sequencing-based surveillance programme for comprehensive early detection of healthcare-associated infection transmission in paediatric oncology patients. J Hosp Infect 2024;143:53–63. https://doi.org/10.1016/j.jhin.2023.10.015.
[20] Xu, X.; Zheng, Y.; Zhang, X.; Zhang, C.; Gai, W.; Yang, Z. Utility of Metagenomic Next-Generation Sequencing for Diagnosis of Infectious Diseases in Critically Ill Immunocompromised Pediatric Patients. Infect. Drug Resist. 2024, 17, 3579–3591. https://doi.org/10.2147/IDR.S472129.
[21]Wu J, Song W, Yan H, Luo C, Hu W, Xie L, et al. Metagenomic next-generation sequencing in detecting path
[22] Lehman AC, Goren LR, Evans MD, Toles O, Drozdov D, Andrews SL, et al. Clinical Performance of Plasma Metagenomic Sequencing in Immunocompromised Pediatric Patients. J Pediatr Infect Dis Soc 2024;13:276–81. https://doi.org/10.1093/jpids/piae024.
[23] Abraham A, Green A, Ferrolino J, Flerlage T, Gowen A, Allison KJ, et al. Utility and Safety of Bronchoalveolar Lavage for Diagnosis and Management of Respiratory Illnesses in

Immunocompromised Children. J Pediatr Infect Dis Soc 2025;14:piaf015.
https://doi.org/10.1093/jpids/piaf015.
[24] Sarana da Silva A, de Campos GM, Altizani GM, de Carvalho E, Barros AC, Cella E, et al. Utilizing Viral Metagenomics to Characterize Pathogenic and Commensal Viruses in Pediatric Patients with Febrile Neutropenia. Viruses 2025;17:345. https://doi.org/10.3390/v17030345.
